# Deciphering and Mitigating Failure Mechanisms in Poly(ether Imide) Corrosion Protection Coatings for Automotive Light-Weighting

**DOI:** 10.1021/acsengineeringau.5c00014

**Published:** 2025-05-16

**Authors:** Tiffany E. Sill, Joseph K. Cantrell, Victor Ponce, Caroline G. Valdes, Torrick Fletcher, Kerry Fuller, Sujata Singh, Mohammed Al-Hashimi, Homero Castaneda, Peter M. Johnson, Sarbajit Banerjee

**Affiliations:** † Department of Chemistry 14736 Texas A&M University, College Station, Texas 77842, United States; ‡ Department of Materials Science and Engineering 14736 Texas A&M University, College Station, Texas 77842, United States; § 114723 SABIC, Mt. Vernon, Indiana 47620, United States; ∥ College of Science and Engineering, 370593 Hamad Bin Khalifa University, P.O. Box: 34110, Doha, Qatar; ⊥ Laboratory for Inorganic Chemistry, Department of Chemistry and Applied Biosciences, 27219 ETH Zürich, CH-5232 Zürich, Switzerland; # Laboratory for Battery Science, Paul Scherrer Institute, Forschunchsstrasse 111, CH-5232 Villigen PSI, Switzerland

**Keywords:** poly(ether imide)s, corrosion protection, coating design, metal binding, aluminum alloys

## Abstract

Corrosion represents a key impediment to the greater adoption of light metal alloys as alternatives to automotive steels in vehicular applications. Thin nanocomposite coatings generate considerable interest for their potential in aluminum alloy corrosion protection, which is challenging due to the lack of conventional protection mechanisms that are available for other metals. Here, we investigate the thickness-dependent corrosion protection afforded to AA 7075 substrates by poly­(ether imide)-based (PEI) coatings. Using electrochemical impedance spectroscopy to monitor ion transport, we observe that with increasing coating thickness, PEI more effectively sequesters ions and enforces permeation selectivity, thereby precluding deleterious substitution processes that dissolve corrosion products. We further explore thickness-dependent modifications to the PEI matrix by incorporation of unfunctionalized exfoliated graphite (UFG) particles to control diffusion processes and co-polymerization with siloxane to manipulate permeation selectivity. Incorporation of UFG platelets can degrade corrosion protection through galvanic coupling with the substrate and enhanced interfacial ion diffusion at lower coating thicknesses. However, interphase development mediated by hydration, network relaxation, and thermal displacement of PEI chains yields a rigid matrix that enhances permeation selectivity and imbues extended tortuosity. This combination results in superior corrosion protection for thicker PEI coatings with embedded UFG platelets under aggressive accelerated corrosion testing conditions. Siloxane co-polymerization, while weakening interfacial adhesion to AA 7075 substrates, facilitates the sequestration of solubilized corrosion products within the matrix under appropriate processing conditions. The results illustrate the importance of understanding the dynamical evolution of polymer secondary structure under aggressive accelerated corrosion testing conditions, point to the specific role of secondary structure and interphasic domains in enforcing permeation selectivity, and establish fundamental thickness limits for retaining effective barrier protection.

## Graphical abstract

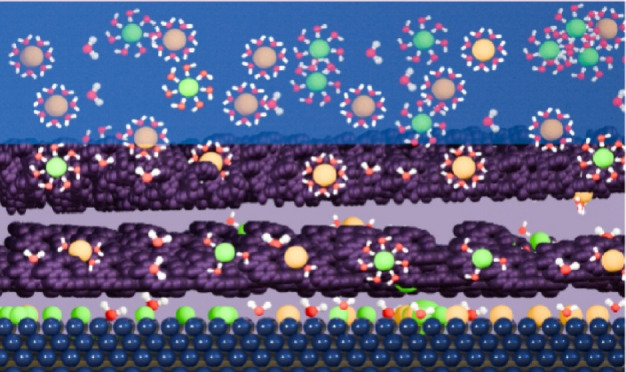

## Introduction

Light-weighting offers a path to reducing fuel consumption and carbon emissions in the transportation sector, an approach that has gained new impetus with the rapid expansion of vehicle electrification.^1–3^ While alloy designs with precisely engineered microstructures enhance the strength of light-weight metals, their use in structural applications exposed to the external environment introduces persistent materials degradation challenges. These issues remain unresolved for some of the most promising candidate materials such as aluminum and magnesium alloys.^4–6^ Localized corrosion in aluminum alloys arises from the galvanic coupling established between the bulk aluminum matrix and microalloyed surface precipitates in corrosive aqueous media, which is exacerbated by chloride-ion-mediated ligand substitution reactions that yield soluble corrosion products.^5^ To mitigate corrosion of light metals, protective coatings employ various mechanisms, including anodic passivation,^7,8^ cathodic protection,^9–11^ barrier protection,^12–14^ and the triggered release of active inhibitors.^15–17^ Typically, nanocomposite coatings integrate multiple corrosion inhibition strategies by incorporating diverse fillers and through finely-tuned modulation of their interphasic interactions within the continuous polymeric matrix.^18^ Poly­(ether imide) (PEI)-based coatings offer best-in-class protection for aluminum alloys, owing to their exceptional interfacial adhesion and unique ability to establish permeation selectivity. Selective permeation arises from the secondary structure formed by polymer chain entanglement, which hinders ion transport to the coating/metal interface.^13,18–20^ In this article, we explore the failure mechanisms of PEI coatings in harsh corrosive environments and examine how targeted modifications, such as co-polymerization and functional filler incorporation, can ameliorate ion permeation to the interface, augment defect tolerance, and mitigate deleterious generation of soluble corrosion products.

In this work, we have focused on corrosion protection of a promising structural material, AA 7075. AA 7075 T6 is a light-weight aluminum alloy commonly used in the aerospace and transportation sectors comprising aluminum, zinc, iron, manganese, chromium, copper, silicon, titanium, and magnesium. PEI frameworks provide exceptional corrosion protection to underlying metal substrates by sequestering ions whilst enabling water permeation. Their outstanding formability ensures conformal coverage of contoured metal surfaces while excellent interfacial adhesion is mediated by Lewis basic interactions between imide carbonyl moieties and Lewis acidic sites on the Al surface.^9,11,13,18–22^ A key advantage of PEI’s ion sequestration and selective permeation, imbued by chain entanglements, is the protection of the aluminum oxyhydroxide passivation layer from chloride-induced substitution reactions. Such barrier protection from ion permeation promotes long-term stability and preservation of the PEI/AA 7075 interface.^13^ Whilst in principle, thicker coatings yield longer, more tortuous diffusion pathways for corrodents to reach the substrate,^23–25^ in practice, processing conditions and surface texturization can imbue defects that deleteriously impact corrosion inhibition.^26–28^

Some important processing conditions for polymer-based coatings include cure/desolvation time, rate of solvent evaporation, deposition and cure temperatures, and rate of coating deposition. The choice of conditions will affect internal and external defect formation, coating homogeneity, chain entanglement, surface texturization, interphase formation, and interfacial adhesion. Processing conditions and coating thickness affect critical film properties including mechanical performance (for instance, brittleness, modulus of elasticity, hardness, impact resistance, and abrasion resistance), optical properties (thicker films provide a greater volume for light–matter interactions), and corrosion resistance. Coating defects such as cracking, cratering (or pinholes), foaming, ribbing flows, and curtain breaks, will degrade coating performance, especially with regards to corrosion protection.^25,29–31^

In harsh corrosive environments, emulated under laboratory conditions through high-temperature exposure to brine or salt-fog, PEI coating failure can occur through multiple mechanisms. Some notable pathways include loss of interfacial adhesion and partial delamination of the coating from the substrate, polymer chain degradation via radical attack, imide hydrolysis, chain disentanglement, and chain scission. Defects can also arise in the macromolecular network of PEI coatings through void formation in fractional free volume near end-caps where permeating species are able to diffuse unincumbered engendering formation of diffusion channels that afford a more direct pathway for ionic diffusion. Ion permeation, water absorption, and compounding effects of localized corrosion resulting from processing imperfections can be further magnified through defective interfaces with fillers that imbue additional pathways for ion transport.^13^ While the resilience of polymeric coatings generally improves with increased thickness because of the greater length of ion diffusion pathways, thicker coatings can also introduce additional scope for imperfections. A critical question that arises is the minimal viable coating thicknesses where desired levels of corrosion protection can be achieved.^25,28^ Such a minimum coating thickness is critical for estimation of economic viability of candidate solutions for automotive lightweighting and for navigating Pareto trade-offs between industrial implementation, cost, and coating longevity.

In this article, we examine the failure mechanisms of PEI coatings with varying thickness upon prolonged exposure to room-temperature brine, high-temperature brine, and salt fog conditions. In order to enhance the longevity of corrosion-resistant PEI coatings, we further examine two distinct approaches to alleviate failure through: (1) inclusion of ion-impermeable unfunctionalized exfoliated graphite as a filler to enhance tortuosity and imbue greater defect tolerance in thick coatings; and (2) co-polymerization of PEI with siloxane moieties to enhance flow properties, obtain pinhole-free thick coatings, and accelerate network relaxation processes.

To evaluate coatings of varying thickness, we specifically utilize electrochemical impedance spectroscopy (EIS) to monitor the evolution of electrochemical behavior over 100 days of immersion in a 3.5 wt % aqueous NaCl solution, 30 days of thermal immersion in 3.5 wt % NaCl solution at 70 °C, and 30 days of ASTM B-117 salt-fog testing. We further investigate structural modifications at the PEI/AA 7075 interface using scanning electron microscopy (SEM) and energy-dispersive X-ray spectroscopy (EDS). Results indicate that unfilled PEI coatings extend the duration of protection as a function of thickness. Thin coatings lack sufficient diffusive bulk to effectively sequester ions, whereas thick coatings tend to contain internal defects that exacerbate failure mechanisms. UFG inclusions elongate ion-diffusion pathways in PEI through interphase development. Copolymerization modifies the PEI backbone to decrease spatial constraints in the polymeric matrix and increase the number of microdeformations during network relaxation, thereby decreasing holidays and pinholes. The results provide valuable insights into the failure mechanisms of multicomponent nanocomposite coatings and inform strategies to ameliorate failure through tailoring interphases and processing conditions.

## Methods

### Synthesis of UFG

UFG was synthesized, as described in previous work.^19,20^ Briefly, 5.0001 g of Bay Carbon SP-1 graphite powder was dispersed in 100 mL of Honeywell Research Chemicals 99.5% purity anhydrous *N*-methyl-2-pyrrolidone (NMP) from Thermo Scientific Chemicals (CAS: 872-50-4) yielding a 4.76 wt % dispersion. The dispersion was then placed in a Branson 5510 ultrasonicator for 8 h to mechanically exfoliate the graphite particles; the sealed, 250 mL borosilicate glass bottle was mechanically agitated every hour to prevent sedimentation of the larger particles and to redistribute particles to improve homogeneity of exfoliation.

### Preparation of UFG/PEI Dispersions

Two types of polymer pellets, provided by SABIC Inc., were dissolved in Honeywell Research Chemicals 99.5% purity anhydrous NMP to achieve a concentration of 10 wt % polymer solution. The two types of pellets were a fully imidized PEI, ULTEM 1000, and a fully imidized siloxane-PEI copolymer, SILTEM 1500. The UFG dispersion was added to a 10 wt % PEI solution of ULTEM 1000 to achieve 3 wt % UFG concentration in the final nanocomposite coating. Additional NMP was added to the dispersion to generate a loading of 5.9 wt % of polymer in NMP, which was previously found to be optimal for spray deposition.^20^ To prevent water absorption, all dispersions were stored under a headspace of nitrogen gas. The dispersions were then spray-coated onto AA 7075 substrates using an automated spray coater (vide infra).

### Size Distribution Analysis of UFG

Size distribution analyses of UFG nanoparticles in the 3 wt % UFG/PEI coating formulation was determined using scanning electron microscopy (SEM) on a FEI Quanta 600 FE-SEM and atomic force microscopy (AFM). UFG/PEI solutions were drop-cast onto silicon wafers and heated on a hot plate temperature of 250 °C to remove residual solvent for SEM and AFM analyses. AFM was performed using a Bruker Dimension Icon AFM. The AFM was completed on tapping mode with a MikroMasch HQ:NSC35 *n*-type silicon tip that had an 8 nm radius, 40° tip cone angle, a force constant of ∼16 N·m^–1^, and a resonance frequency of 300 kHz. Images were analyzed on NanoScope Analysis 2.0 software. The SEM images and AFM maps, presented in Supporting Figure S1, demonstrate the relatively homogenous distribution of UFG nanoparticles within the 3 wt % UFG/PEI coating samples. TEM images of 3 wt % UFG/PEI are provided in Supporting Figure S2.

### AA 7075 Substrate Preparation

Aluminum clad alloy, AA 7075 T6, was purchased from Bralco Metals then cut into 10 cm × 10 cm square substrates. The composition of AA 7075 is provided in Table 1 below.^19^ One side of the square substrate was uniformly abraded with P100 grit sandpaper, then washed with hexanes (UN1208, Fisher Chemical), before a final acetone (UN1090, Fisher Chemical) rinse to prepare the metal substrates for spray coating.^13,20^

**Table 1:** Nominal Chemical Composition in Weight % (wt %) of T-6 AA 7075 Substrates

Zn	Mg	Cu	Si	Cr	Fe	Mn	Ti	Al
5.1–6.1	2.1–2.9	1.2–2.0	0–0.4	0.18–0.28	0–0.5	0–0.3	0–0.2	balance

### Spray Coating

Three types of coatings were deposited: PEI as a control, a siloxane-PEI copolymer (denoted hereon as Si-PEI),^32,33^ and a 3 wt % unfunctionalized exfoliated graphite (UFG) inclusion in PEI nanocomposite coating (UFG/PEI).^20^ Three different thicknesses of coatings were spray-coated, under identical processing conditions, onto AA 7075 substrates from NMP solutions. The thin coatings ranged between 4 and 7 μm, the middle thickness ranged between 11 and 16 μm, and the thickest coatings ranged between 20 and 30 μm coating thicknesses as measured using a Dr. NIX Byko-Test 8500 Basic thickness meter. A Specialty Coating Systems Precisioncoat V automatic spray coating machine, retrofitted with a hot plate and polytetrafluoroethylene tubing, was used to spray coat the substrates. The automatic spray coating system enables control and optimization of the spray-coating parameters: rate of coating deposition, spray droplet size, rate of NMP evaporation, radius of the spray cone, particle acceleration, and substrate impact. Control over the aforementioned parameters influences the conformity, homogeneity, thickness, and structure of the molecular network of the resulting coatings. As such, the automatic spray coating system enables mitigation of visible void-space and pinholes in the coating samples.^20^ The hot plate was set to 475 °C, which yielded a temperature range of 220–410°C throughout the duration of the spray coating process, as verified using a handheld VWR High Temperature InfraRed Thermometer. Substrates were placed on the hot plate for 5 min and pre-equilibrated to temperatures of 390–410°C prior to engaging the spray coating profile. Lower temperatures in the range were measured during spray deposition as the air flow and room temperature solution cooled the substrates momentarily. Between deposition passes, a 30 s pause was added to allow residual solvent to evaporate fully prior to depositing the next layer. The spray nozzle had an orifice diameter of 0.7112 cm and was placed at a *z*-axis height of 12.065 cm, which generated a spray cone with a diameter of 2.286 cm. The feed rate was maintained at 0.407 mL/min, and the atomization pressure of 9.0 kPa was held constant across all coating samples produced for the study. A total of 15–20 passes were applied to yield coatings devoid of visible pinholes with thickness ranging from 20 to 30 μm.^13,20^

### EIS

EIS measurements were performed over the course of 100 days by immersing the coated substrates in an aerated 3.5 wt % aqueous NaCl solution held at ca. 20–25 °C to examine the degradation of the coatings. EIS measurements were further performed on coated substrates subject to 30 days of immersion in 3.5 wt % brine solution at 70 °C, and coated substrates subject to ASTM B-117 salt-fog exposure. The electrochemical cell used a flat glass O-ring flange placed on top of an O-ring and pinch clamped onto the PEI-coated substrate to isolate a working electrode with a surface area of 5.226 cm^2^. The reference electrode was a saturated calomel electrode (SCE) from Gamry, and the counter electrode was a Pt/Nb mesh soldered to a Nb rod. The electrochemical cell was placed within a Faraday cage. Each EIS measurement was preceded by an OCP measurement for 10 min, followed by potentiostatic impedance spectroscopy in the frequency range of 100 kHz to 10 mHz with ten points/decade and an amplitude of ±10 mV. Each test was repeated on duplicate substrates. Collected EIS data was fitted to equivalent circuit models using Gamry EChem Analyst software and Pine Research AfterMath software.

The AC impedance response is plotted as Bode and Nyquist plots. The Bode plot indicates the magnitude of the impedance with respect to frequency. The overall impedance is contrasted at the lowest frequency, |*Z*|_0.01Hz_. Our previous work has revealed that the overall impedance of the bare AA 7075 substrate is around 10^5^ Ω/cm^2^ with two discernible time-constants.^13,20^ Charge-transfer processes are captured in the capacitive frequency region located at 10^3^–10^–1^ Hz. Deposition, absorption, and adsorption processes are identified in the inductive region in the frequency range of 10^–1^–10^–2^ Hz.^13,20,34,35^

### Circuit Fit Modeling for Determination of Coating Capacitance

After EIS measurements were converted into Bode and Nyquist plots, circuit fit modeling was performed on each of the corresponding EIS plots. Circuit fit modeling, determined by evaluation of the number of time constants inferred from Bode and Nyquist plots, provides a means of decoupling electrochemical processes based on their distinctive frequency-dependent response. Capacitance values were extrapolated from the circuit fit models, which were generated using Gamry Analyst software or Pine Research AfterMath software.^36,37^ The models provide a goodness-of-fit parameter, which is generated to minimize residual errors, preferably on the order of 10^–3^ or lower. In circuit fit modeling of organic coatings, substantial noise can result from high water retention, or as a result of stochastic fluctuations, if the system has not reached steady-state. As a result, Kramer-Kronig analyses were performed to ensure the data collected represented the intrinsic response of the coating samples. No data points were excluded in our circuit fit models and a noise induction element^35^ has not been used.^38^

Coating capacitance is expressed using eq 1:

$$C_{c}=\frac{{\varepsilon \varepsilon }_{0}A}{t}$$ 1

where *A* is the testing area, *t* is the coating thickness, ε is the dielectric constant of the medium, and ε_0_ is the free space permittivity in vacuum. The dielectric constant of both PEI and Si-PEI is ca. 3.02,^39,40^ as compared to a value of 78.4 for water.^41^ Owing to the contribution of water to the cumulative dielectric constant, water absorption into the polymer increases the dielectric constant, thereby increasing the measured capacitance. The increase in capacitance modeled using EIS provides a sensitive probe of water uptake.^13^

### Open Circuit Potential (OCP) Measurements

A three-electrode system was utilized where the coated substrates operated as the working electrode.^13,19,20^ OCP measurements were performed for a total time of 600 s at a sampling period of 0.5 s using a saturated calomel electrode as the reference electrode, a Pt/Nb mesh counter electrode, and the coated substrate as the working electrode across a sample area of 5.226 cm^2^. OCP values were plotted to determine the corrosion potential and interpret water uptake in the coating over the course of the study.^13,19,20,34^

### Adhesion Testing

Three types of standardized American Society of Testing and Materials (ASTM) testing were performed. Reported tests performed include the ASTM D2197-13 scrape test.^42^ The ASTM D3359-17 tape test^43^ was performed; however, all samples pre-exposure and post-exposure resulted in a 5B classification and will not be further discussed. Similarly, the ASTM D4541-17 pull-off test^44^ demonstrated that all samples exceeded the strength of the epoxy provided in the standardized test kit and will also not be further discussed.^13^

### Cross-Sectional Imaging of Coating/Substrate Interface

Cross-sectional SEM images were acquired for pre-exposure, as-coated samples, and coated substrates post-exposure to three separate tests including 100 days of immersion in a 3.5 wt % aqueous solution of NaCl, 30 days of immersion in 3.5 wt % aqueous NaCl solution at elevated temperatures of 70 °C, and the ASTM B-117 ^45^ 30 day salt-fog exposure to a 5 wt % aqueous NaCl solution. Changes in coating thickness and the coating/substrate interface were examined by SEM imaging and EDS mapping of aluminum. Substrates with a diameter of 2.54 cm were punched out and immersed in an EpoxiCure 2 epoxy resin and hardener mixed in a 4:1 (w/w) ratio and allowed to harden for 24 h. Sections for cross-sectional imaging were cut in half using a Buehler IsoMet diamond precision saw. Half of the sample was subsequently ground on a grinding/polishing wheel (Buehler EcoMet 30) with 1200 grit P600, and subsequently, 4000 grit P1200 silicon carbide sandpaper. The sample was next polished using a 1 μm water-based diamond suspension (Electron Microscopy Sciences, CAT# 50372-41), on 200 mm polishing pads (Struers MD-Floc). Samples were then coated with 5 nm of Pt/Pd using a Ted Pella Cressington 108 Sputter Coater prior to imaging by SEM.^13,20^

### Field-Emission SEM

SEM imaging was performed on a TESCAN-LYRA and a JEOL JSM-7500F ultra-high-resolution field-emission instrument with a low-aberration conical objective lens, and a cold cathode UHV field-emission conical anode gun. SEM images were acquired at a working distance of 8 mm, accelerating voltage of 20.0 keV, an emission current of 20 μA, and a probe current set at 10 nA/cm^2^. An Oxford EDS system equipped with X-ray and digital imaging was used for elemental mapping of cross-sectioned samples.^13,20^

### Transmission Electron Microscopy (TEM)

TEM was performed using a JEOL JEM-2010 instrument with an accelerating voltage of 200 kV. The TEM sample was prepared using a Formvar-coated 400 mesh copper TEM grid. 3 wt % UFG/PEI dispersion was drop-cast onto the copper grid to obtain an electron-beam-transparent coating. The grid was then placed on a hot plate set to 200 °C to remove residual solvent.

## Results

### Thickness-Dependent Corrosion Performance of PEI-Coated AA 7075

A critical descriptor for the efficacy of corrosion protection of Al alloys by polymer coatings is the ability of the coatings to prevent Cl-ion migration and attack at the coating/Al interface. Reaction with Cl-ions transforms insoluble aluminum oxyhydroxide passivation layers into more soluble chloride-substituted corrosion products, which are gradually dissolved and transported along a concentration gradient to the coating surface and then into the electrolyte, resulting in localized corrosion.^5^ PEI shows exceptional interfacial adhesion to AA 7075 substrates mediated by Lewis acid–base interactions between imide carbonyl moieties and Lewis acidic Al surface sites.^20^ To gain insight into barrier protection offered by coatings in a variety of environments, three different accelerated exposure tests have been performed on coated AA 7075 samples, 100 day immersion in a 3.5 wt % aqueous NaCl solution; 30 day immersion in a 3.5 wt % NaCl aqueous solution at 70°C; and 30 days of ASTM B-117 salt-fog testing in 5 wt % aqueous NaCl solution.

EIS has been performed to monitor the electrochemical evolution of PEI-coated substrates with varying thicknesses subject to 100 days of immersion in a 3.5 wt % aqueous NaCl solution. Figure 1 shows data acquired for 4–7 μm (thin), 11–16 μm (medium), and 20–30 μm (thick) PEI-coatings on AA 7075. SEM has been employed to image the physical coating/metal interface and EDS mapping to generate elemental Al maps to examine metal migration. The ion transport resistance of PEI coatings increases monotonically with increasing coating thickness corresponding to greater diffusion lengths that must be traversed by corrodents to reach the substrate interface. As such, the initial overall impedance at |*Z*|_0.01Hz_ are ca. 10^7^ Ω/cm^2^ for thin, ca. 10^9^ Ω/cm^2^ for intermediate thickness, and ca. 10^10^ Ω/cm^2^ for thick coatings. Figure 1g shows that for the thin coatings under consideration, measured impedance is variable over the 100 day immersion, ranging between |*Z*|_0.01Hz_ of ca. 10^8^ Ω/cm^2^ on day 14 to |*Z*|_0.01Hz_ of ca. 10^6^ Ω/cm^2^ on day 75, then ultimately returning to |*Z*|_0.01Hz_ of 10^7^ Ω/cm^2^ by day 100. The diminishing radii of capacitive loops present in the Nyquist plot, Figure 1j, indicate an increase in the rate of charge transfer events that occur at the coating/metal interface, which suggest continuous breakdown and restoration of the passive layer, as well as pit nucleation and propagation. Increasing the thickness of the coatings in Figure 1h,i yields greater retention of overall coating impedance although pronounced deterioration is nevertheless observed in Figure 1c, and corresponding Al EDS map, Figure 1e, even for the thickest 20–30 μm PEI-coated AA 7075 substrate. As such, Figure 1 illustrates that thicker coatings yield monotonically improved corrosion protection of AA 7075 but the magnitude improvement of corrosion protection is lower than anticipated, and that thicker coatings nevertheless suffer from pitting corrosion on prolonged exposure. The observed lack of linear scaling is posited to arise from the greater concentration of imperfections in thicker coatings. Increased coating thickness under similar coating specifications increases the probability of defects that can engender localized failure. For instance, solvent evaporation rates are governed by thermal gradients established across thick coatings. As such, solvent elimination occurs more rapidly from interfacial layers closer to the heat source as compared to the coating surface. As a result, the sub-layers will begin to rapidly cross-link within localized regions, which in turn generates stress gradients across the coating.^25,29,31^ In thicker coatings where a greater amount of solvent is remnant in the upper layers, blisters, voids, or pinholes can be generated upon eventual solvent elimination. Pinholes resulting from incomplete or inhomogeneous solvent removal from thicker coatings can serve to engender localized uptake of corrodent species and can enable formation of diffusion channels. Furthermore, pit propagation can be accelerated as a result of localized acidic zones manifested inside pinholes.^34^

**Figure 1:**
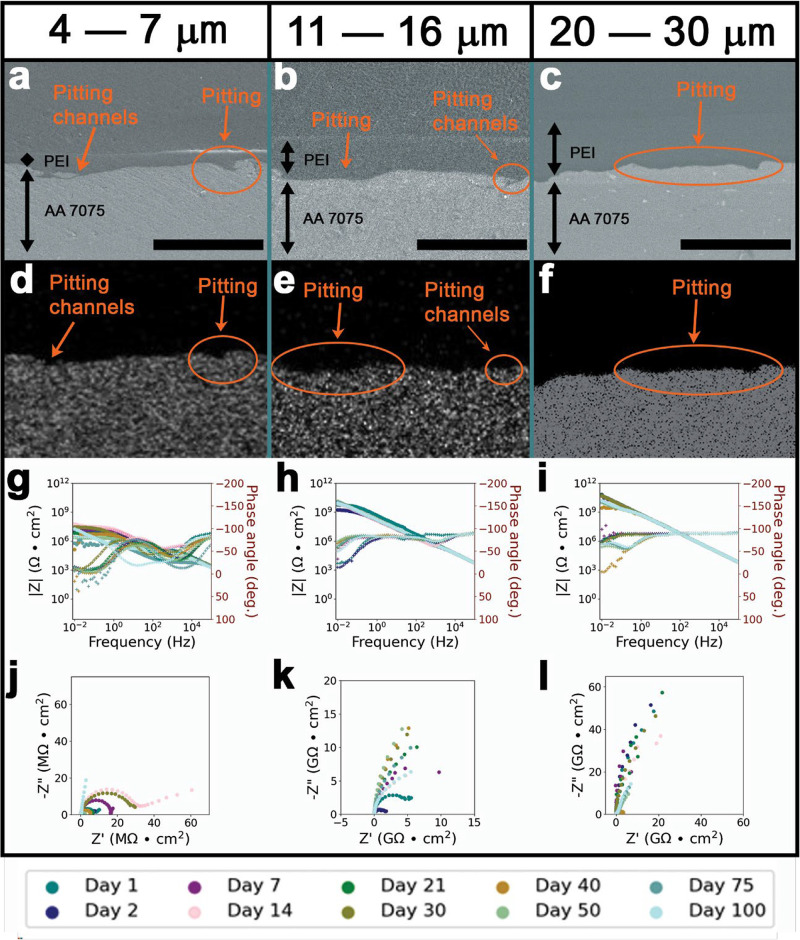
Thickness-dependent corrosion performance of PEI coatings. Post-exposure SEM cross-sectional view of (a) 4–7 μm; (b) 11–16 μm; and (c) 20–30 μm on AA 7075 substrates after 100 days of exposure to 3.5 wt % aqueous solutions of NaCl. The scale-bars correspond to 50 μm. Aluminum EDS maps of AA 7075 substrates coated with (d) 4–7 μm; (e) 11–16 μm; and (f) 20–30 μm. Bode plots corresponding to AA 7075 substrates coated with (g) 4–7 μm; (h) 11–16 μm; and (i) 20–30 μm. Nyquist plots for (j) 4–7 μm; (k) 11–16 μm; and (l) 20–30 μm monitored across 100 days of exposure to 3.5 wt % aqueous solutions of NaCl.

Two specific modifications are examined in subsequent sections as a means of increasing defect tolerance: (a) incorporation of UFG to enhance the effective tortuosity of ion diffusion pathways^19,20^ and to thus alleviate loss of permeation selectivity upon PEI degradation; and (b) co-polymerization with siloxanes to improve rheological properties (melt flow rate of 12 g/10 min of at 295 °C/6.6 kgf of Si-PEI versus 9 g/10 min at 337 °C/6.6 kgf for PEI) and thereby reduce the concentration of pinholes and holidays in the polymeric matrix. Si-PEI exhibits a ca. 50 °C decrease in glass transition temperature (*T*_g_) upon co-polymerization as compared to PEI, which is expected to enhance chain mobility and abet annealing of defects during spray deposition. While imperfections remain inevitable, whether resulting from processing conditions or induced by external factors (e.g., chain scission, hydrolysis, void formation) involved in deleterious corrosion events, increasing defect tolerance windows can significantly increase coating lifetimes resulting in extended protection and delaying or preventing catastrophic failure.

### Coating Imperfections and Their Implications for Corrosion Inhibition Afforded by Thin PEI Coatings

To first discuss the effects of modifications for 4–7 μm, thin, coatings, upon 100 days of immersion in a 3.5 wt % aqueous NaCl solution and 30 days of ASTM B-117 salt-fog exposure in 5 wt % brine solution, Figure 2 shows that overall UFG/PEI and Si-PEI fare relatively worse than PEI alone (Figure 1 and Supporting Figure S3). Examining the dynamical evolution of the EIS response provides insight into some nuances. The 4–7 μm UFG/PEI (Figure 2i) demonstrates superior performance to the unmodified PEI (Figure 1g) until day 100. On day 75, both the PEI and UFG/PEI systems diverge out of steady-state, thereby precluding an accurate EIS measurement.^34,35^ The unfilled PEI subsequently undergoes network relaxation processes and returns to steady-state conditions by day 100, whereas the UFG/PEI does not, which points to slower network relaxation dynamics in UFG/PEI, which is attributed to greater rigidity, likely derived from interphase formation (vide infra).^13,46,47^ SEM images of the 4–7 μm UFG/PEI-coated substrates in Figure 2a,c, and corresponding EDS aluminum maps shown in Figure 2e,g show pit propagation in both corrosion environments, 100 day immersion and salt-fog respectively, with localized coating swelling in pitted regions. However, the degradation is not severe enough to cause a significant change in adhesion (Table 2).

**Figure 2:**
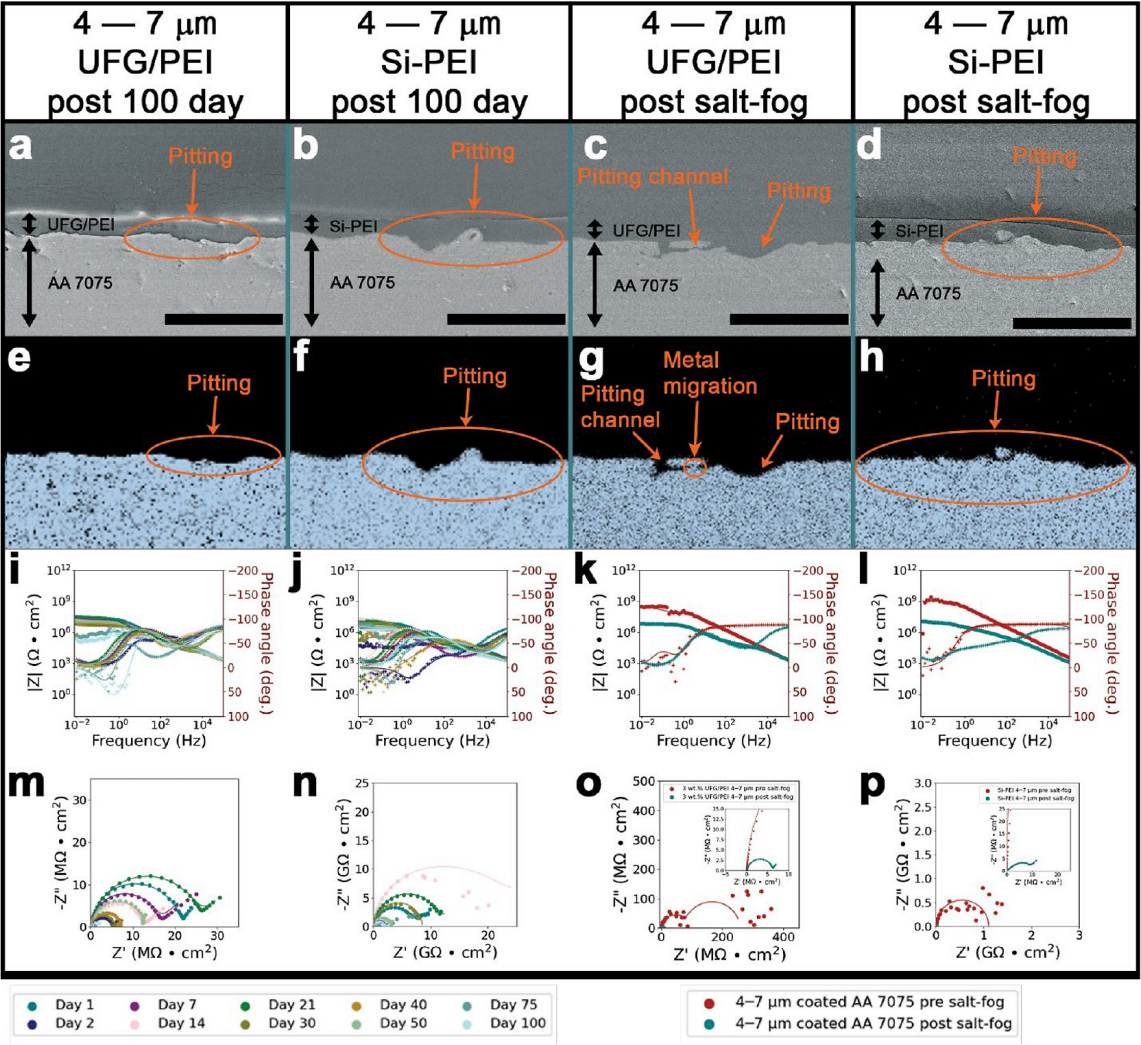
Corrosion Protection Afforded by Thin UFG/PEI and Si-PEI Coatings. Post-exposure SEM cross-sectional view of (a) 4–7 μm 3 wt % UFG/PEI; and (b) 4–7 μm SI-PEI on AA 7075 substrates after 100 days of exposure to 3.5 wt % aqueous solutions of NaCl. Post-exposure SEM cross-sectional view of (c) 4–7 μm 3 wt % UFG/PEI; and (d) 4–7 μm SI-PEI on AA 7075 substrates after 30 days of ASTM B-117 salt-fog exposure to 5 wt % aqueous solutions of NaCl. The scale-bars correspond to 50 μm. Aluminum EDS maps of AA 7075 substrates coated with (e) 4–7 μm 3 wt % UFG/PEI; and (f) 4–7 μm Si-PEI after 100 days of exposure to 3.5 wt % aqueous solutions of NaCl. Aluminum EDS maps of AA 7075 substrates coated with (g) 4–7 μm 3 wt % UFG/PEI; and (h) 4–7 μm SI-PEI after 30 days of ASTM B-117 salt-fog exposure to 5 wt % aqueous solutions of NaCl. Bode plots corresponding to AA 7075 substrates coated with (i) 4–7 μm 3 wt % UFG/PEI; and (j) 4–7 μm Si-PEI over 100 days of immersion in 3.5 wt % aqueous NaCl solutions at ambient conditions. Bode plots corresponding to AA 7075 substrates coated with (k) 4–7 μm 3 wt % UFG/PEI; and (l) 4–7 μm Si-PEI after 30 days of ASTM B-117 salt-fog exposure in 5 wt % aqueous NaCl. Nyquist plots for (m) 4–7 μm 3 wt % UFG/PEI; and (n) 4–7 μm Si-PEI coated AA 7075 substrates monitored across 100 days of exposure to 3.5 wt % aqueous solutions of NaCl. Nyquist plots for (o) 4–7 μm 3 wt % UFG/PEI; and (p) 4–7 μm Si-PEI coated AA 7075 substrates monitored across 30 days of ASTM B-117 salt-fog exposure to 5 wt % aqueous solutions of NaCl.

**Table 2:** ASTM Scrape Adhesion Testing Results for the Three Different Thickness Variations of Si-PEI and UFG/PEI Coatings on AA 7075 Substrates As-Cast and after Corrosion Exposure to 3.5 wt % Aqueous Solutions of NaCl for 100 Days, Thermal 3.5 wt % Brine Solutions at 70 °C for 30 Days, and ASTM B-117 Salt-Fog Testing for 30 Days

test/sample	ASTM D2197-13 (scrape test)
PEI	
4–7 μm as-cast	>10.0 kg
4–7 μm post 100 day	8.60 kg
4–7 μm post thermal	6.30 kg
4–7 μm post salt-fog	6.40 kg
11–16 μm as-cast	>10.0 kg
11–16 μm post 100 day	9.20 kg
11–16 μm post thermal	7.20 kg
11–16 μm post salt-fog	7.20 kg
20–30 μm as-cast	>10.0 kg
20–30 μm post 100 day	>10.0 kg
20–30 μm post thermal	9.50 kg
20–30 μm post salt-fog	>10.0 kg
Si-PEI	
4–7 μm as-cast	4.25 kg
4–7 μm post 100 day	4.05 kg
4–7 μm post thermal	6.00 kg
4–7 μm post salt-fog	4.05 kg
11–16 μm as-cast	5.85 kg
11–16 μm post 100 day	5.95 kg
11–16 μm post thermal	7.55 kg
11–16 μm post salt-fog	6.10 kg
20–30 μm as-cast	8.55 kg
20–30 μm post 100 day	7.55 kg
20–30 μm post thermal	7.70 kg
20–30 μm post salt-fog	6.50 kg
UFG/PEI	
4–7 μm as-cast	7.50 kg
4–7 μm post 100 day	6.05 kg
4–7 μm post thermal	7.80 kg
4–7 μm post salt-fog	7.00 kg
11–16 μm as-cast	>10.0 kg
11–16 μm post 100 day	8.00 kg
11–16 μm post thermal	8.70 kg
11–16 μm post salt-fog	7.80 kg
20–30 μm as-cast	>10.0 kg
20–30 μm post 100 day	>10.0 kg
20–30 μm post thermal	>10.0 kg
20–30 μm post salt-fog	>10.0 kg

The Bode plot (Figure 2i) of the 4–7 μm UFG/PEI-coated AA 7075 shows an initial |*Z*|_0.01Hz_ value of ca. 10^8^ Ω/cm^2^ and is characterized by three time-constants. Two additional time-constants are manifested by day 30 when the impedance value is decreased by an order of magnitude. Subsequently, on day 75, the |*Z*|_0.01Hz_ value is reduced to ca. 10^5^ Ω/cm^2^, which persisted for the remainder of the 100 day immersion period. The Bode plot (Figure 2k), corresponding to the 4–7 μm UFG/PEI-coated AA 7075 that was subjected to ASTM B-117 salt-fog exposure demonstrates a reduction in overall impedance by two orders of magnitude. The observed salt-fog performance is a result of the incorporation of greater free volume through creation of additional UFG/PEI interfaces, wherein aerosolized salt water microdroplets can permeate through imperfections and be transported to the PEI/AA 7075 interface. The Nyquist plot (Figure 2m) demonstrates diminishing radii of capacitive loops further indicating degradation of the AA 7075 (see also the equivalent circuit fit models in Figure S4 and Table S1). The relatively inferior performance of UFG/PEI observed in Figure 2, as compared to PEI alone (Figure 1), is attributable to galvanic coupling between UFG particles and the AA 7075 surface. Such galvanic coupling leads to a percolative network that accelerates corrosion^19,48–50^ and enables migration of Al to the coating surface, as is clearly observed in Figure 2a,c. In addition, the UFG incorporation creates a new interface within the PEI matrix, which can give rise to additional free volume within the matrix and promote localized ion diffusion. For 4–7 μm thin coatings, UFG/PEI thus affords less control over the corrosion product as compared to PEI alone.

The SEM images (Figure 2b,d) and corresponding aluminum EDS maps (Figure 2f,h) for the 4–7 μm Si-PEI-coated substrates analogously shows severe pitting failure accompanied by a nominal loss of adhesion (Table 2). Coating swelling is further observed in Figure 2b, which is most prominent above the areas of pit propagation and almost doubles the pre-exposure coating thickness over the 100 day immersion period. Notably, the 4–7 μm Si-PEI-coated AA 7075 samples that were exposed to 100 days of brine immersion and 30 days of salt-fog testing displayed the lowest measured adhesion out of all the samples tested. The Bode plot for the 4–7 μm Si-PEI-coated substrate (Figure 2j) depicts a low initial overall impedance value of |*Z*|_0.01Hz_ of ca. 10^6^ Ω/cm^2^, which is decreased by two orders of magnitude over the first 30 days of exposure, then increased back to the original |*Z*|_0.01Hz_ value of ca. 10^6^ Ω/cm^2^ by day 50; this value is retained with some fluctuations for the remainder of the 100 day immersion study. Similarly, the post salt-fog exposure 4–7 μm Si-PEI-coated-AA 7075 shows an initial impedance of |*Z*|_0.01Hz_ ca. 10^9^ Ω/cm^2^, which is deteriorated to a final impedance value of |*Z*|_0.01Hz_ ca. 10^6^ Ω/cm^2^ (Figure 2l). These results suggest that the more aggressive salt-fog testing exacerbates internal coating defects, which leads to greater deterioration of the Si-PEI molecular network in thinner coatings. While the Si-PEI coating is expected to enable more rapid network relaxation processes, accelerated vapor and aerosol permeation through thin Si-PEI coatings results in rapid corrosion of the AA 7075 substrate upon salt-fog exposure. The Bode plots in Figure 2j,l further exhibit variability in the number of kinetic time-constants, which range between 2 and 5 features over the course of both exposure conditions. The Nyquist plots for the 4–7 μm Si-PEI-coated substrates (Figure 2n,p) are characterized by diminishing capacitive loops indicating an increase in the occurrence and rate of charge-transfer events. Equivalent circuit models for the 100 day brine immersion of the varied thickness Si-PEI-coated AA 7075 samples are presented in Figure S5; see also Table S2 for a description of which circuit fit models correspond to each day of the corrosion exposure. As such, for 4–7 μm thin coatings, Si-PEI also fares worse than PEI, which is attributed to worse interfacial adhesion resulting from co-polymerization with siloxane monomers, which do not bind Lewis acidic sites on the AA 7075 substrate. Consistent with this hypothesis, Table 2 shows that the thickest 20–30 μm PEI-coated AA 7075 demonstrates higher adhesion (greater than 10.0 kg) as compared to the 20–30 μm Si-PEI-coated counterpart prior to exposure. These results corroborate that the excellent interfacial adhesion of PEI to AA 7075 substrates is disrupted by co-polymerization with siloxane groups, which degrades corrosion protection afforded by PEI alone (Figure S3). As such, both types of modifications, UFG inclusions and siloxane co-polymerization, degrade the performance of PEI alone for thin coatings. We next examine whether such modifications, with appropriate processing, can increase the defect tolerance of thicker PEI coatings.

### Facilitating Interphase Development in UFG/PEI Coatings

Figure S1 shows the size distribution of UFG inclusions, which is consistent with extensive previous analyses.^19^ Figure S2 shows TEM images exemplifying the homogeneous dispersion of UFG particles in PEI. Pronounced differences are observed for all three coating thicknesses upon contrasting post thermal exposure coating samples of UFG/PEI (presented in Figure 3) and PEI (shown in Figure S6), which were immersed in 3.5 wt % aqueous NaCl solutions at an elevated temperature of 70 °C for 30 days. The representative Bode plot in Figure 3g for 4–7 μm UFG/PEI reveals the overall impedance was reasonably maintained, within an order of magnitude, at |*Z*|_0.01Hz_ ca. 10^7^ Ω/cm^2^. Only two time-constants are discernible over the full 30 day period of thermal exposure for 4–7 μm UFG/PEI-coated AA 7075. In contrast, impedance (Figure S6g) of 4–7 μm PEI-coated substrate fluctuates around an average value of |*Z*|_0.01Hz_ ca. 10^5^ Ω/cm^2^, indicating the superior performance of UFG/PEI coatings under aggressive thermal conditions. Diminishing radii of the capacitive loops, evident in the respective Nyquist plot (Figure 3j) for the thin UFG/PEI coating, reflect a modest decline in performance (see also the equivalent circuit-fit models in Figure S7 and Table S3 for 30 day thermal exposure). In contrast, the Nyquist plot (Figure S6g) for the unfilled PEI coating equivalent reveals the presence of an induction loop and a substantial amount of noise, which is most likely attributed to water absorption. As such, corrosion protection in thermal environments is markedly improved for UFG/PEI as compared to PEI alone, and further, is more pronounced with increasing coating thickness (comparing Figures 3 and S6). While some isolated pitting is observed in SEM images (Figure 3b,c) and EDS maps (Figure 3e,f) for 11–16 μm medium and 20–30 μm (thick) UFG/PEI coated substrates, the post-mortem SEM image in Figure S6c illustrates substantially greater pitting present in the thermally exposed 20–30 μm PEI-coated AA 7075. Adhesion is somewhat diminished for the 11–16 μm UFG/PEI-coated substrate following 30 days of immersion under thermal conditions (Table 2) but the adhesion of the 20–30 μm thick UFG/PEI coating is preserved within limits of measurement.

**Figure 3:**
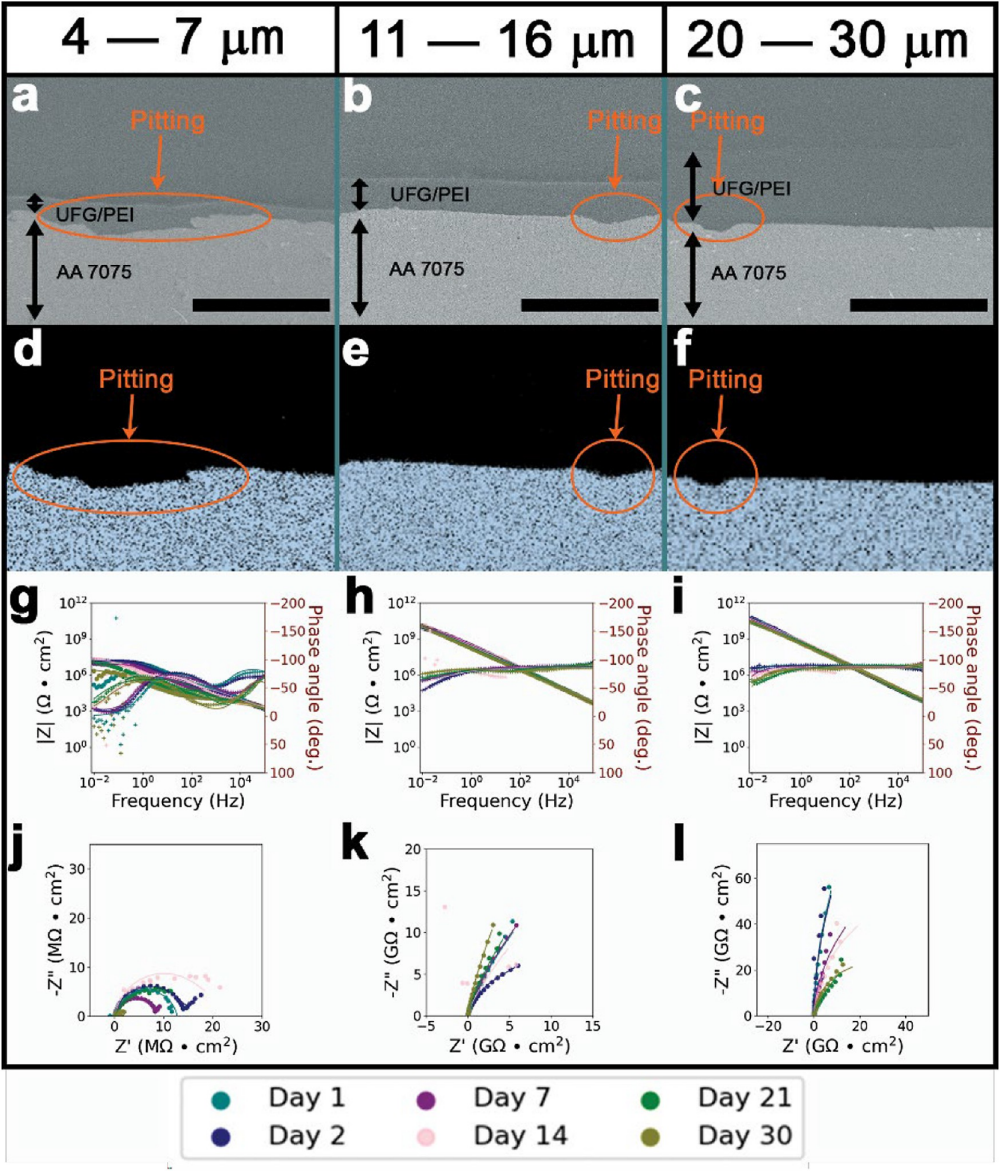
Corrosion performance of UFG/PEI coatings on AA 7075 after 30 days of immersion in 3.5 wt % aqueous NaCl at 70 °C. Post-exposure SEM cross-sectional view of (a) 4–7 μm 3 wt % UFG/PEI; (b) 11–16 μm 3 wt % UFG/PEI; and (c) 20–30 μm 3 wt % UFG/PEI on AA 7075 substrates after 30 days of thermal exposure to 3.5 wt % aqueous solutions of NaCl at 70 °C. The scale-bars correspond to 50 μm. Aluminum EDS maps of AA 7075 substrates coated with (d) 4–7 μm UFG/PEI; (e) 11–16 μm UFG/PEI; and (f) 20–30 μm UFG/PEI. Bode plots corresponding to AA 7075 substrates coated with (g) 4–7 μm UFG/PEI; (h) 11–16 μm UFG/PEI; and (i) 20–30 μm UFG/PEI. Nyquist plots for (j) 4–7 μm UFG/PEI; (k) 11–16 μm UFG/PEI; and (l) 20–30 μm UFG/PEI monitored across 30 days of thermal exposure to 3.5 wt % aqueous solutions of NaCl at 70 °C.

The impedance of the intermediate thickness 11–16 μm UFG/PEI-coated AA 7075 substrate, maintained at |*Z*|_0.01Hz_ ca. 10^10^ Ω/cm^2^, as observed in the Bode plot (Figure 3h) is characterized by a single time-constant for the entire 30 day exposure period. The capacitive behavior depicted in the Nyquist plot (Figure 3k) and equivalent circuit models (Figure S7 and Table S3) suggest excellent preservation of barrier protection. In contrast, the electrochemical impedance response of the bare 11–16 μm PEI-coated substrate (Figure S6h,k) reveals a reduction in overall impedance values, by as much as three orders of magnitude, and contraction of capacitive loop radii. Similarly, the Bode plots for thermal exposure of the 20–30 μm thick coating (Figure 3i) is characterized by a single time-constant and a sustained |*Z*|_0.01Hz_ of ca. 10^11^ Ω/cm^2^ across the entire period of exposure. Nyquist plots (Figure 3l) for the sample demonstrate near-ideal capacitive behavior (see also Figure S7 and Table S3), which is further characteristic of superior barrier protection. The thickest PEI-coated AA 7075 demonstrates equivalent progression in overall impedance (Figure S6i), but the smaller radii of capacitive loops revealed by the Nyquist plot (Figure S6l) corroborates the enhanced protection afforded by the UFG/PEI coating formulation under aggressive thermal exposure conditions. The improved performance of the nanocomposite coatings is ascribed to the formation of interphasic domains through π–π interactions between the PEI framework and the basal planes of UFG upon sustained heating. The π–π stacking of PEI aromatic rings and π-conjugated basal planes has been widely documented in the literature including in our previous work.^21,51–53^ Raman spectroscopy results evidence non-covalent π–π interactions between carbon nanofillers and PEI based on downshifts of the UFG D-band at ∼1350 cm^–1^, G-band at ∼1580 cm^–1^, and 2D-band at ∼2700 cm^–1^.^19,54^ The π–π interactions anchor polymer chains to the active interfaces of the nanoparticles at UFG/PEI boundaries, which yields a pronounced interphase region. The interphase region minimizes fractional free volume, impedes ion transport, and enhances mechanical properties, and has been directly manifested through amplified hardness, increased fracture toughness, improved thermal stability, more efficient load transfer, and a reduction of microcrack formation.^55–59^ Interphase development can be demonstrated through adhesion testing and nanoindentation. Nanoindentation in past studies confirmed a significant increase in elastic modulus and mean hardness for samples that contained UFG loadings below the percolation-threshold.^13^ The 3 wt % UFG/PEI formulation, used in this study, was previously determined to exhibit considerable viscoelastic character, which is suggestive of most extensive interphase formation.^20^ The π–π interactions and the resulting formation of a robust interphase between the PEI-polymer chains and homogeneously dispersed UFG nanoparticles gives rise to enhanced tortuosity and reduced ion diffusion while still allowing adequate spatial mobility for network relaxation within the nanocomposite matrix.^19,20,60^ As a result of hydration and rearrangement of polymer chains in the molecular network supplanting interfacial free volume with rigid interphasic domains upon prolonged heating in the thermal exposure measurements, UFG inclusions greatly enhance tortuosity of ion diffusion pathways and yield sustained barrier protection.^13,20^

Since interphases such as observed in UFG/PEI coatings cannot be formed for Si-PEI coatings, pitting corrosion is further exacerbated in thermal environments for thin and intermediate thicknesses as shown in Figure S8a,b, respectively. Under thermal conditions, the 4–7 μm Si-PEI-coated AA 7075 has an initial |*Z*|_0.01Hz_ value of ca. 10^6^ Ω/cm^2^ that decays rapidly (Figure S8g), and by day 2 of the exposure period the |*Z*|_0.01Hz_ value of ca. 10^4^ Ω/cm^2^ remains constant, implying a complete loss of protection (worse than PEI alone, Figure S6g) under thermal exposure conditions. Intriguingly, the 4–7 μm Si-PEI-coated AA 7075 substrate displays a significant increase in adhesion after thermal exposure conditions (Table 2), which is likely a result of additional siloxane crosslinking to the corrosion product.^61^ The equivalent circuit models for the 30 day thermal exposure of the three thicknesses of Si-PEI coated substrates are shown in Figure S9 (see also Table S4). The thick Si-PEI-coated substrates are discussed in a subsequent section.

### Increased Defect Tolerance in Thick Si-PEI Coatings

We next evaluate whether the greater flowability imbued by the rheological properties of Si-PEI endows improved corrosion protection at higher thicknesses. Figure S10 presents the intermediate thickness, 11–16 μm, Si-PEI and UFG/PEI coated samples. The SEM images (Figures S8b and S10b,d) and corresponding aluminum EDS maps (Figures S8e and S10f,h) establish that moderate pitting is present upon corrosion exposure under all conditions. Coating expansion is localized around pits in the 11–16 μm Si-PEI-coated substrates after 100 days of immersion at room temperature (Figure S8b), accompanied by a nominal increase in adhesion. However, SEM images of the 11–16 μm Si-PEI coating samples after 30 days of both thermal immersion (Figure S8b) and salt-fog testing (Figure S10d) exhibit substantial uniform swelling, in conjunction with considerable improvement in adhesive strength (Table 2), as noted in Figure S8 of the Supporting Information. The Bode plot in Figure S10h displays an initial |*Z*|_0.01Hz_ value of ca. 10^8^ Ω/cm^2^ and is typified by four time-constants. Conversely, on day 7, the overall impedance increased to |*Z*|_0.01Hz_ of ca. 10^10^ Ω/cm^2^, which suggests that the system has reached steady-state. Over the remainder of the 100 day immersion study, the impedance displays moderate instability characterized by deviation in time-constants and impedance ranging between |*Z*|_0.01Hz_ of ca. 10^9^–10^10^ Ω/cm^2^. As such, the coating performance is improved over thin Si-PEI coatings shown in Figure 2j,l but is nevertheless inferior to PEI alone (Figure 1h).

The thick 20–30 μm Si-PEI coatings manifest greater defect tolerance, exceeding the performance of PEI alone under all exposure conditions (contrast Figures 4 and S8 to data for thick PEI coatings in Figures 1, S3 and S6). The EIS evolution observed in the Bode plots in Figure 4e,f exemplify the excellent barrier performance of the 20–30 μm Si-PEI-coated substrates with a measured impedance of |*Z*|_0.01Hz_ ca. 10^11^ Ω/cm^2^, similarly characterized by a two time-constants, which are maintained throughout the entire 100 day immersion in 3.5 wt % brine solution and 30 day ASTM B-117 salt-fog exposure to 5 wt % aqueous NaCl, respectively. The Nyquist plot for the 20–30 μm Si-PEI-coated substrate subject to 100 day brine immersion, Figure 4g, suggests the sample behaves as a near-ideal capacitor indicating the electrochemical activity of the system remains constant and limited over the exposure period (see also Figure S5 and Table S2). While the Bode plot for the 30 day thermal exposure of the thick 20–30 μm Si-PEI-coated AA 7075 (Figure S8i) does contain multiple time-constants, as evidenced in the equivalent circuit-fit models (Figure S9 and Table S4) of the Nyquist plot (Figure S8l), the overall impedance is similarly maintained at a value of |*Z*|_0.01Hz_ ca. 10^11^ Ω/cm^2^. The apparent reduction in the radius of the capacitive loop visible in the Nyquist plot in Figure 4h suggests an increase in the rate of charge-transfer events at the coating/metal interface resulting in some pit nucleation and propagation over the 30 day salt-fog exposure period. SEM imaging (Figures 4a,b and S8c) of the AA 7075 substrates coated with 20–30 μm Si-PEI, indeed, revealed the greatest severity of pitting was present in the sample that was exposed under aggressive salt-fog conditions. The post-mortem SEM images of all 20–30 μm Si-PEI coated samples demonstrate some degree of pit formation, indicating some metal dissolution is prevalent under all of the exposure conditions. However, the aluminum EDS maps of the 20–30 μm Si-PEI-coated substrates (Figures 4c,d and S8f) show relatively modest pitting. These results imply that the oxidized metal migrating out of the substrate is predominantly captured within the Si-PEI network and retained close to the coating/metal interface, thus mitigating diffusion pathways via chain rearrangement. The accumulation of metal at the interface is further evidenced through the reduction in adhesive strength exhibited by all the 20–30 μm Si-PEI samples (Table 2). Collectively, the imaging, adhesion, and EIS evolution indicate that with thicker Si-PEI coatings, the improved flow properties localize defects and prevent transport of soluble corrosion products from the interface, thereby preserving corrosion performance over extended periods.

**Figure 4:**
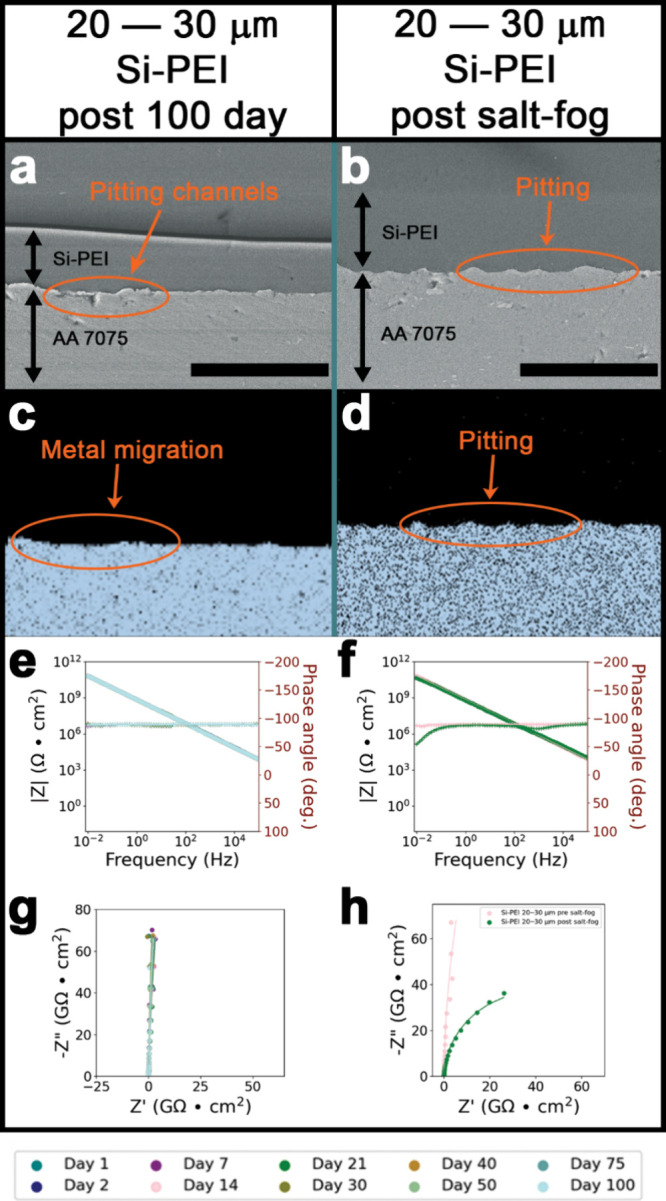
Corrosion Performance of Thick Si-PEI coatings on AA 7075. Post-exposure SEM cross-sectional view of (a) 20–30 μm SI-PEI on AA 7075 after 100 days of exposure to 3.5 wt % aqueous NaCl solution. Post-exposure SEM cross-sectional view of (b) 20–30 μm SI-PEI on AA 7075 after 30 days of ASTM B-117 salt-fog exposure to a 5 wt % aqueous solution of NaCl. The scale-bars correspond to 50 μm. Aluminum EDS map of AA 7075 substrates coated with (c) 20–30 μm Si-PEI after 100 days of exposure to 3.5 wt % aqueous brine solution. Aluminum EDS map of AA 7075 coated with (d) 20–30 μm SI-PEI after 30 days of ASTM B-117 salt-fog exposure to a 5 wt % aqueous solution of NaCl. Bode plot corresponding to AA 7075 coated with (e) 20–30 μm Si-PEI over 100 days of immersion in 3.5 wt % aqueous NaCl solution under ambient conditions. Bode plot corresponding to an AA 7075 substrate coated with (f) 20–30 μm Si-PEI after 30 days of ASTM B-117 salt-fog exposure in 5 wt % aqueous NaCl. Nyquist plot for (g) 20–30 μm Si-PE-coated AA 7075 monitored across 100 days of exposure to 3.5 wt % aqueous NaCl. Nyquist plot for (h) 20–30 μm Si-PEI-coated AA 7075 monitored across 30 days of ASTM B-117 salt-fog exposure to a 5 wt % aqueous solution of NaCl.

### Mechanistic Origins of Thickness-Dependent Corrosion Inhibition

The preceding sections provide experimental evidence for the evolution of corrosion resistance in PEI, UFG/PEI, and Si-PEI coatings on AA 7075 as a function of thickness. In general, barrier protection increases with coating thickness under all accelerated corrosion conditions. Figure 5 portrays the evolution of calculated coating capacitance for 20–30 μm PEI, UFG/PEI, and Si-PEI coatings in the 100 day immersion (Figure 5a) and 30 day thermal exposure studies, which are governed by structure-dependent water absorption. The high barrier protection evinced for the thick 20–30 μm Si-PEI-coated substrate over 100 day brine immersion is a result of substantial network relaxation. Initial coating saturation is reached almost immediately on day 2, illustrated by the capacitance value of ca. 10^–7^ Ω^–1^·cm^–2^·s*^n^*, which is followed by a two orders of magnitude decrease by day 14. As a result of microdeformations and chain rearrangement during the relaxation processes, the coating absorbs more water, which yields a second saturation point on day 50. Further plastic deformation and network relaxation of the 20–30 μm Si-PEI coating is manifested by a decrease in coating capacitance over the remaining 50 days reaching ca. 10^–9^ Ω^–1^·cm^–2^·s*^n^* by day 100. There is a single observed saturation point in the 20–30 μm unfilled PEI coating at day 50 where the calculated capacitance increased almost two orders of magnitude. The lower energetic and spatial constraints in the secondary structure generated by the aliphatic siloxane co-polymer present in the Si-PEI coating system permits greater mobility of the macromolecular network as compared to relatively more rigid PEI alone.^13,62–64^ Water uptake data reported for the two resins indicates that PEI accommodates more water molecules, in deionized water, as compared to Si-PEI (1.3% saturation *versus* 0.5% saturation respectively)^39,40,65^ ; however, the coating samples demonstrate that Si-PEI retains more water than PEI (Figure 5a). This indicates that the coefficient of water expansion is greater in Si-PEI than in PEI alone resulting from greater chain mobility. The greater amount of void space in the polymeric matrix of Si-PEI yields higher diffusion rates, which is indeed observed as immediate saturation. Consequently, a higher number of charge-transfer events can be activated, resulting in more severe corrosion of the underlying metal substrate. The Si-PEI coatings capture aluminum ions migrating away from the metal interface and generate domains of sequestered corrosion products (Figure 4). The capacitance corroborates those findings as the newly developed domains fill void space, thereby impeding continuous hydration of the sub-layers closer to the interface. This observed phenomenon results in water retention in the upper layers of the thicker Si-PEI coatings.^66^ As such, the thicker Si-PEI coatings enforce permeation selectivity and impede ion transport; however, at lower thicknesses, the accelerated water absorption and extensive water retention rapidly degrades coating performance.

**Figure 5:**
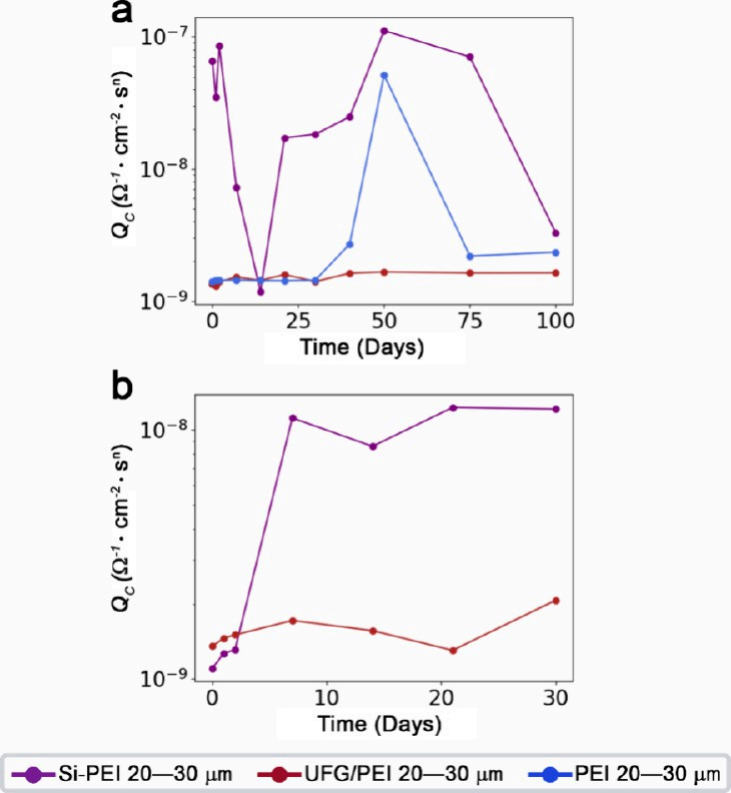
Contrasting coating capacitance modulation resulting from water absorption. Coating capacitance of the AA 7075 substrates coated with 20–30 μm Si-PEI, UFG/PEI, and unfilled PEI after (a) 100 days of immersion in 3.5 wt % aqueous NaCl solution at ambient temperatures; and (b) 30 days of immersion in 3.5 wt % brine solution at elevated temperatures of 70 °C.

In contrast, the thick 20–30 μm UFG/PEI coatings maintain a constant capacitance value of ca. 10^–9^ Ω^–1^·cm^–2^·s*^n^*, evident in both the 100 day immersion (Figure 5a, see also Figure S11) and the 30 day immersion under thermal conditions (Figure 5b), indicating minimal water absorption into the polymeric matrix. Indeed, consistent with this notion, the ASTM adhesion tests in Table 2 evidence that UFG/PEI-coated samples maintain higher adhesion than their Si-PEI counterparts at the same thickness.^20,67^

Figure 6 provides a schematic illustration of a thinner coating (Figure 6a) with a relatively short, and direct, diffusion length for ions to traverse before reaching the PEI/AA 7075 interface. Upon reaching the aluminum surface, Cl-ions initiate a ligand-exchange reaction transforming the passivating aluminum oxyhydroxide layer (*K*_sp_ of 1.8 × 10^–5^ in pure water, near 25 °C) to a more soluble aluminum oxychlorohydroxide intermediate corrosion product. The aforementioned chloride-ion ligand-exchange reaction propagates until all the oxygen and hydroxyl groups are replaced culminating in the formation of the highly soluble tetrachloroaluminate anion corrosion product (*K*_sp_ of 20,400 in pure water, near 25 °C).^13,20,34,68–70^ In contrast, thicker coatings afford longer, more tortuous ion-diffusion pathways through entangled polymer coils (Figure 6b); such entanglements constitute an amorphous glassy matrix that sequesters ionic species and slows corrosion processes.^13,47,71–73^

**Figure 6:**
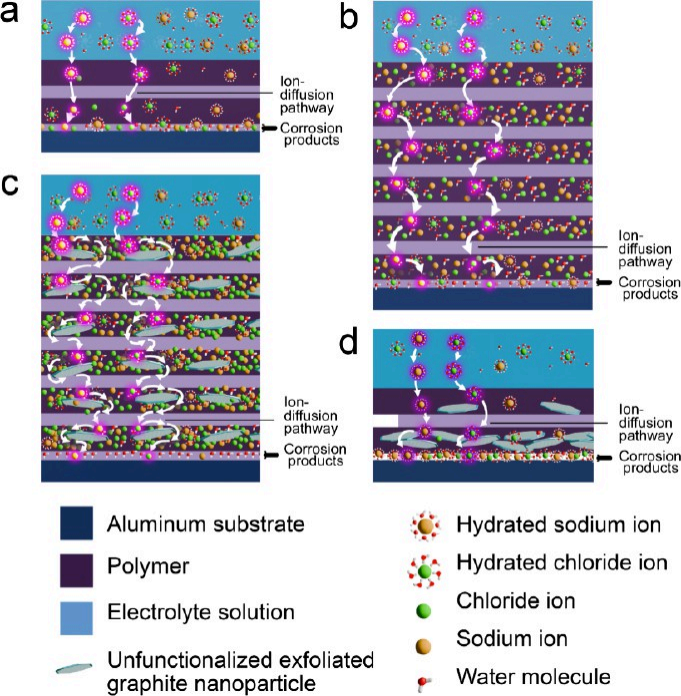
Mechanistic origins of thickness-dependent corrosion performance and composite modifications to PEI to enhance barrier protection. (a) Schematic illustration of a thin, 4–7 μm PEI coating on AA 7075. The resulting matrix has shorter ion-diffusion path lengths and has a limited active volume to sequester ions, which allows the relative facile transport of corrodents from the electrolyte to the coating/metal interface where they participate in deleterious charge-transfer events; Cl-ions reaching the substrate initiate pitting corrosion and solubilize the corrosion product based on ligand-exchange reactions. (b) Transport processes in a thicker 20–30 μm PEI coating on AA 7075; longer ion-diffusion pathways and a greater active volume enables more effective ion sequestration, resulting in greater resistance to ion transport from the electrolyte to the coating/substrate interface; thus, the integrity of the insoluble corrosion product is preserved. (c) Increased tortuosity and greater effective length of ion-diffusion pathways engendered through incorporation of UFG particles. UFG incorporation yields a more rigid matrix because of interphase formation that enables permeation of water but sequesters ions and precludes them from reaching the interface. As such, interfacial adhesion is preserved, and the corrosion product is not solubilized. (d) Thin coatings deposited via spray coating concentrate UFG particles at the coating metal/interface and do not develop a clear interphase. This yields a percolative network, additional interfaces, and free volume that promotes ion transport to the interface thereby accelerating deleterious corrosion processes.

Nanocomposite designs incorporate functional filler materials to increase defect tolerance windows and mitigate common failure modes to maintain tensile strength, prevent stress cracking through enhanced load transfer, and prevent decomposition of polymers through incorporation of fillers that foster interphase development.^74,75^ Upon the incorporation of UFG within the continuous matrix (Figure 6c) and as a result of chain hydration, network relaxation, and thermally activated displacements (such as during prolonged testing under thermal conditions), a rigid interphase region is generated, mediated by π–π interactions between the heterocyclic PEI groups and the π-conjugated basal planes of UFG. The interphase domains further sequester ions within the molecular network.^20,56,57,76^ Being themselves impermeable to ions, UFG inclusions and their surrounding interphases enhance the tortuosity of ion diffusion pathways in the polymeric matrix, and thereby enhance barrier protection.^19,20,59,77^ Note that for UFG to be effective in enhancing the defect tolerance of PEI coatings, the formation of an optimal interphase is imperative. Past work including our extensive finite element numerical simulations has demonstrated that the size distribution of UFG particles is critical to interphase development and the overall reduction of fractional free volume within amorphous nanocomposite matrices.^19^ Upon mechanical exfoliation of unfunctionalized graphite, particles are fractured, thereby decreasing the lateral dimensions of resultant particles. Size distribution analyses in our previous work revealed that particles ranging from ca. 350–200 nm in lateral dimensions constitute the majority proportion of UFG particles present in UFG/PEI coatings.^19^ The SEM and AFM images in Figure S1 are consistent with our previous results. Such particles develop a robust interphase region and engender less fractional free volume surrounding the filler particles within the coating.^19,78^ Void space adjacent to UFG particles are internal defects that can cause two detrimental effects on the corrosion performance of the coating. First, voids provide more direct diffusion pathways for corrodents to reach the substrate; and second, the open surface area on the UFG nanoparticles affords sites for parasitic reactions such as polymer and graphite oxidation.^19,78,79^ As such, in order to increase defect tolerance windows in UFG/PEI coatings, it is of pivotal importance to fabricate nanocomposites as demonstrated in Figure S2 with a thick enough polymer region to fully enrobe UFG particles, preclude formation of a percolative matrix of particles, and mitigate voids at the polymer/filler interface. For thin coatings, UFG particles situated in close proximity of the AA 7075 substrate can generate a galvanic couple and enhance corrosion, which is further exacerbated by anion diffusion and transport of solubilized corrosion products through remnant free volume at graphene/PEI interfaces (Figure 6d).^19,48–50^

Thicker Si-PEI coatings fare worse than UFG/PEI but afford enhanced corrosion protection at high thicknesses as compared to PEI alone by dint of their facile access to network relaxation processes that yield entangled networks that can maintain permeation selectivity and localize transport of corrodent species to the interface and solubilized corrosion products from the interface.^66^

## Conclusions

Light-weighting vehicular components is predicated on the design of resilient, protective coatings that preserve underlying multicomponent Al alloys from corrosion upon exposure to saline and hypersaline environments across a broad temperature range. In this study, we explore failure mechanisms of PEI corrosion protective coatings of AA 7075 as a function of thickness and upon incorporation of a UFG filler and a siloxane co-polymer. Thin 4–7 μm coatings all exhibit substantial pitting corrosion of the underlying aluminum substrate. Indeed, the base PEI polymer surpasses both the filler and co-polymer modifications in performance by dint of its superior interfacial adhesion. In contrast, when examining thicker coatings, 20–30 μm UFG/PEI-coated AA 7075 shows exceptional corrosion inhibition including under aggressive salt-fog conditions and under high-temperature immersion in brine. The outstanding corrosion performance of UFG/PEI is particularly pronounced at higher temperatures when network relaxation and chain mobility engender an optimal interphase anchoring polymer chains to UFG basal planes through π–π interactions. The rigid interphase and surrounding interlocked PEI chains selectively sequester ions and ensure preservation of oxyhydroxide passivation products at the PEI/AA 7075 interface. Si-PEI coatings, whilst somewhat inferior to UFG/PEI, afford improved performance as compared to PEI alone at high thicknesses. The facile network relaxation and greater chemical stability of Si-PEI coatings engenders the ability to block ion transport and imbues greater defect tolerance. As such, optimal coating design requires interfacial adhesion governed primarily by choice of the baseline polymer, which in the case of PEI is enabled by strong Lewis acid–base interactions between imide carbonyl moieties and Al surface sites in AA 7075. However, to ensure long-term resilience in aggressive environments, protective coatings require the establishment and preservation of tortuous ion diffusion pathways and the ability to selectively sequester and limit the mobility of ions and solubilized corrosion products. Future work will explore multilayered systems with appropriate surface treatment and interfacial adhesion with PEI, followed by coating of a subsequent layer implementing strategies to limit the mobility of ions and solubilized corrosion products. Future work will furthermore explore direct imaging of the interphase structure through cryo electron focused ion beam sectioning and cryo-transmission electron microscopy.

## Supplementary Material



## Data Availability

Data will be made available upon request.

## Notes

SEM and AFM images of UFG nanoparticle inclusions; TEM images of 3 wt % UFG/PEI coatings; SEM images, Al EDX maps, Bode plots, and Nyquist plots for unfilled PEI coatings after 30 days of ASTM B-117 salt-fog exposure; equivalent circuit fit models for all three thickness variants of 3 wt % UFG/PEI coatings after 100 days of immersion in 3.5 wt % aqueous NaCl solution; table correlating equivalent circuit fit models for all three thickness variants of 3 wt % UFG/PEI coatings after 100 days of immersion in 3.5 wt % aqueous NaCl solution; equivalent circuit fit models for all three thickness variants of Si-PEI coatings after 100 days of immersion in 3.5 wt % aqueous NaCl solution; table correlating equivalent circuit fit models for all three thickness variants of Si-PEI coatings after 100 days of immersion in 3.5 wt % aqueous NaCl solution; SEM images, Al EDX maps, Bode plots, and Nyquist plots for unfilled PEI coatings after 30 days of immersion in 3.5 wt % NaCl at elevated temperatures; equivalent circuit fit models for all three thickness variants of 3 wt % UFG/PEI coatings after 30 days of immersion in 3.5 wt % aqueous NaCl solution at elevated temperatures; table correlating equivalent circuit fit models for all three thickness variants of 3 wt % UFG/PEI coatings after 30 days of immersion in 3.5 wt % aqueous NaCl solution at elevated temperatures; SEM images, Al EDX maps, Bode plots, and Nyquist plots for Si-PEI coatings after 30 days of immersion in 3.5 wt % NaCl at elevated temperatures; equivalent circuit fit models for all three thickness variants of Si-PEI coatings after 30 days of immersion in 3.5 wt % aqueous NaCl solution at elevated temperatures; table correlating equivalent circuit fit models for all three thickness variants of Si-PEI coatings after 30 days of immersion in 3.5 wt % aqueous NaCl solution at elevated temperatures; SEM images, Al EDX maps, Bode plots, and Nyquist plots for intermediate thickness coatings of 3 wt % UFG/PEI and Si-PEI coatings after 30 days of immersion in 3.5 wt % NaCl at elevated temperatures; and SEM images, Al EDX maps, Bode plots, and Nyquist plots for 20–30 μm thicknesses of 3 wt % UFG/PEI coatings after 100 days of immersion in 3.5 wt % NaCl aqueous solution and 30 days of ASTM B-117 salt-fog immersion (PDF)
