# Supplementary material for: Deciphering and Mitigating Failure Mechanisms in Poly(ether Imide) Corrosion Protection Coatings for Automotive Light-Weighting
Source: ACS Eng Au. 2025 May 16;5(4):384–99. doi: 10.1021/acsengineeringau.5c00014 (PMC12371726; doi:10.1021/acsengineeringau.5c00014)
Supplement: Supplementary file 1 [file eg5c00014_si_001.pdf]

Supporting Information

Deciphering and Mitigating Failure Mechanisms in Polyetherimide Corrosion Protection Coatings for Automotive Light-weighting

Tiffany E. Sill,[1,2] *Joseph K. Cantrell,*[1,2] *Victor Ponce,*[2] *Caroline G. Valdes,*[1] *Torrick Fletcher Jr,*[1] *Kerry Fuller,*[3] *Sujata Singh,*[1,2] *Mohammed Al-Hashimi,*[4] *Homero Castaneda,*[2] *Peter M. Johnson,*[3]* *and Sarbajit Banerjee*[1,2,5,6]*

*P.M.J. and S.B. are equally corresponding authors this paper.

[1]Department of Chemistry Texas A&M University, College Station, Texas 77842, United States. [2]Department of Materials Science and Engineering Texas A&M University, College Station, Texas 77842, United States. [3]SABIC, Mt. Vernon, Indiana 47620, United States. [4]College of Science and Engineering, Hamad Bin Khalifa University, P.O. Box: 34110, Doha, Qatar. [5]Laboratory for Inorganic Chemistry, Department of Chemistry and Applied Biosciences, ETH Zürich, CH-5232, Zürich, Switzerland. [6]Laboratory for Battery Science, Paul Scherrer Institute, Forschunchsstrasse 111, CH-5232 Villigen PSI, Switzerland.

Correspondence: Peter M. Johnson (peter.johnson@sabic-hpp.com), Sarbajit Banerjee (sbanerje@ethz.ch)

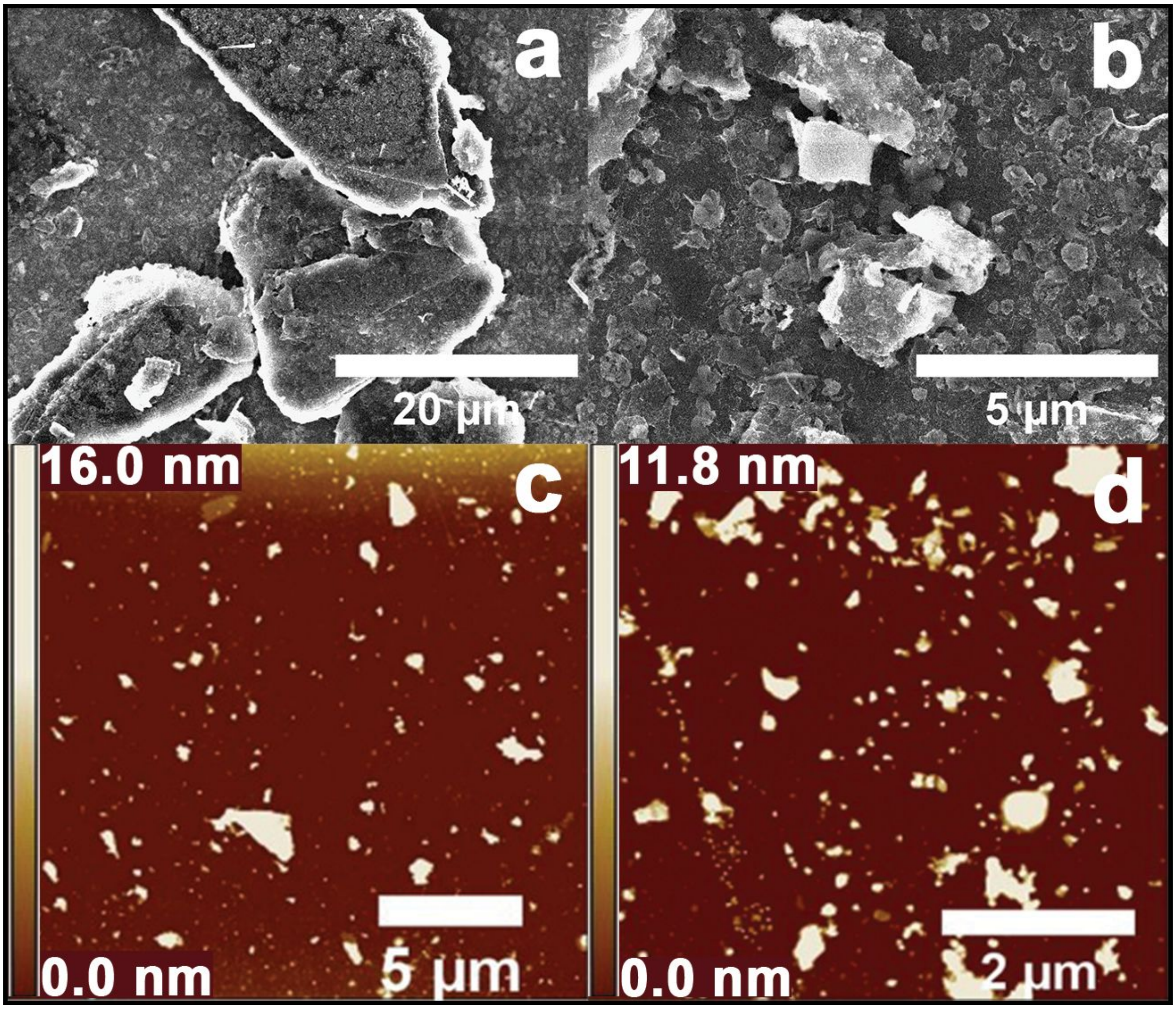

Figure S1: Size and distribution determination for UFG nanoplatelets in 3 wt. % UFG/PEI nanocomposite coatings. *a,b) SEM images acquired for UFG particles incorporated into 3 wt.% UFG/PEI coating formulations at varying magnifications. c,d) AFM images exhibiting the UFG particle distribution and thickness The dimensions of UFG particles is consistent with our previously published analyses.*[1]

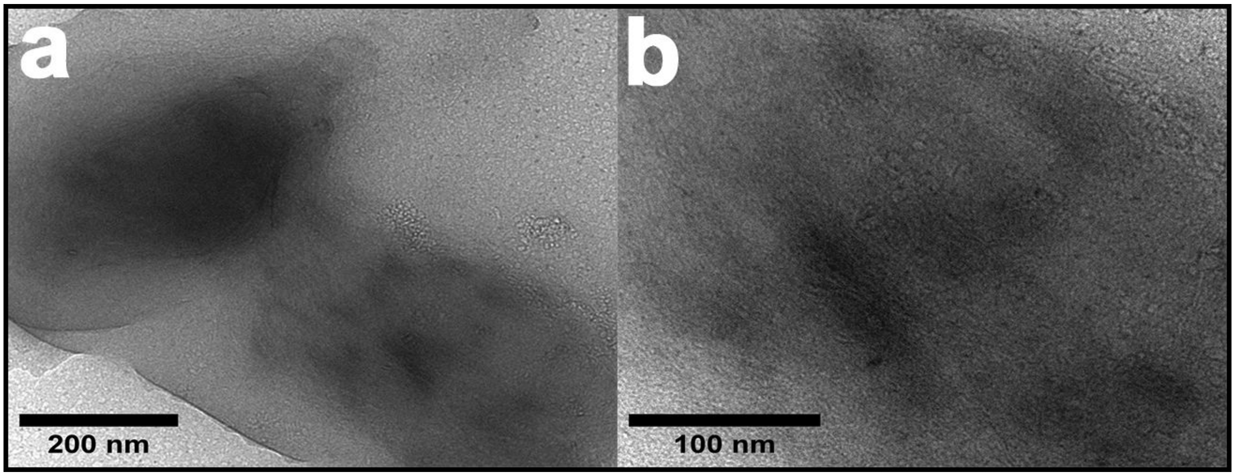

Figure S2: TEM image of 3 wt.% UFG/PEI demonstrate the dispersion of UFG particles within the nanocomposite coating. *The darker regions in the image presenting a greater electron density contrast are the UFG particles. The TEM images demonstrate the excellent dispersion of UFG particles within the PEI matrix.*

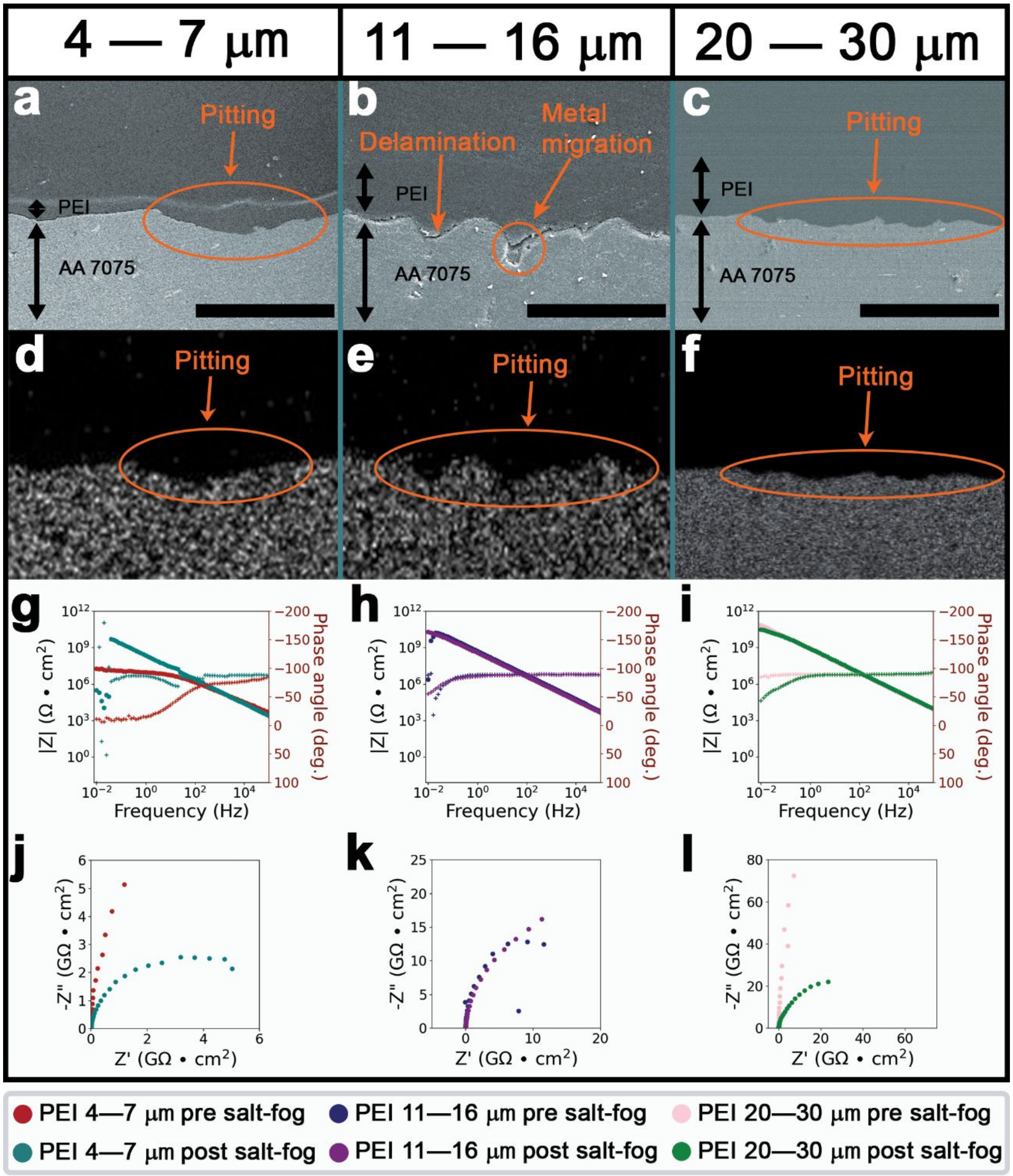

Figure S3: Corrosion protection afforded by varying thicknesses of PEI coatings in a salt fog environment. *Post-exposure SEM cross-sectional view of a) 4—7 μm; b) 11—16 μm; and c) 20—30 μm PEI on AA 7075 substrates after 30 days of ASTM B-117 salt-fog exposure in 5 wt.% aqueous solutions of NaCl. The scale-bars correspond to 50 μm. Aluminum EDS maps of AA 7075 substrates coated with d) 4—7 μm PEI; e) 11—16 μm PEI; and f) 20—30 μm PEI. Bode plots corresponding to AA 7075 substrates coated with g) 4—7 μm PEI; h) 11—16 μm PEI; and i) 20—30 μm PEI. Nyquist plots for j) 4—7 μm PEI; k) 11—16 μm PEI; and l) 20—30 μm PEI monitored across 30 days of ASTM B-117 salt-fog exposure in 5 wt.% aqueous solutions of NaCl.*

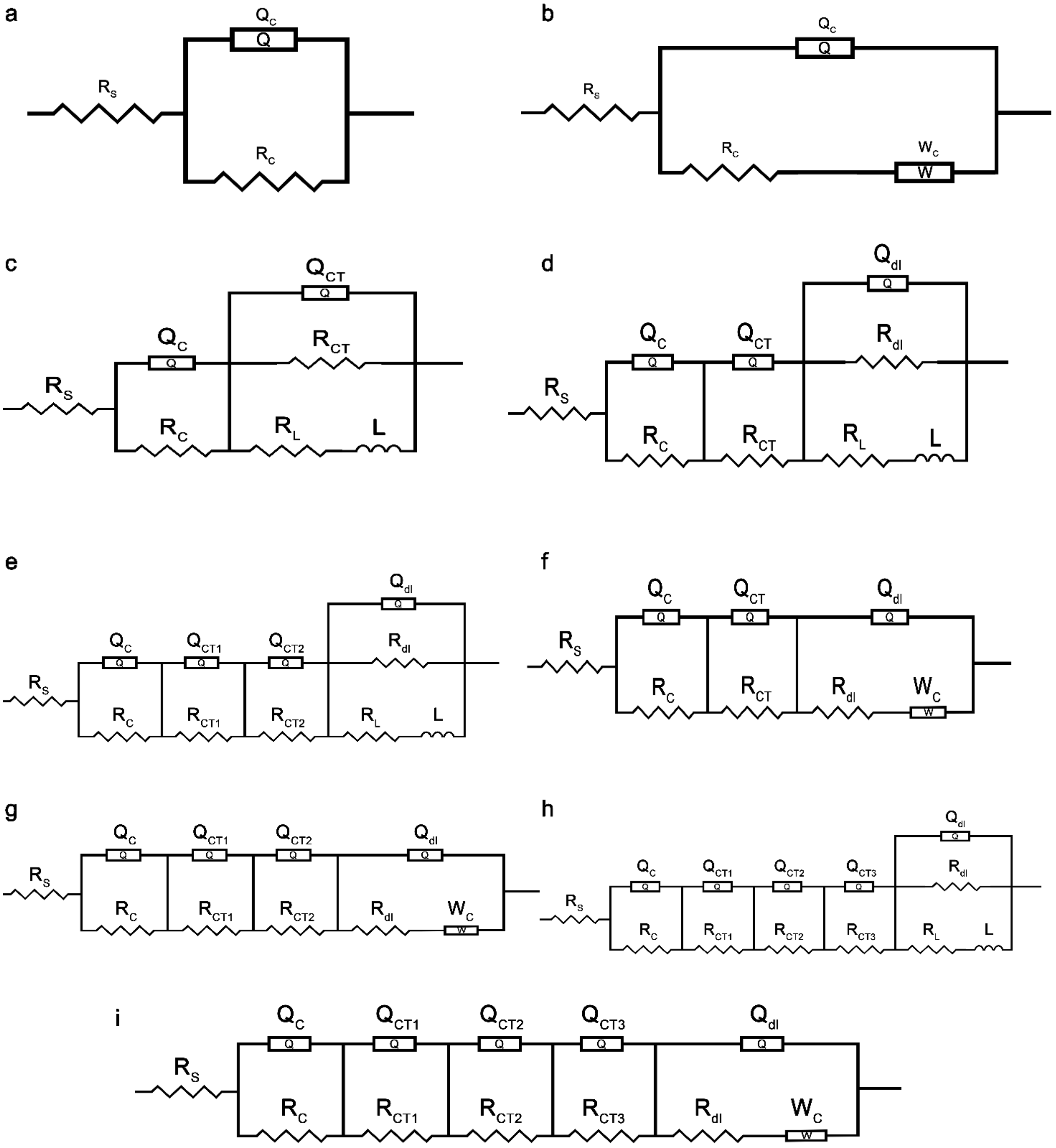

Figure S4: Equivalent circuits modeling the evolution of the impedance response of AA 7075 substrates coated with 4—7 µm 3 wt.% UFG/PEI, 11—16 µm 3 wt.% UFG/PEI, and 20—30 µm 3 wt.% UFG/PEI then immersed in a 3.5 wt.% aqueous solution of NaCl for 100 days at ambient conditions. *a) represents a single Randles cell; b) depicts a single Randles cell with a Warburg diffusion element; c) portrays one Randles cell in series with an inductor; d) illustrates two Randles cells in series with an inductor; e) shows three Randles cells in series with an inductor; f) displays three Randles cells in series with a Warburg diffusion element; g) demonstrates four Randles cells in series with a Warburg diffusion element; h) illustrates four Randles cells in series with an inductor; and i) describes five Randles cells in series with a Warburg diffusion element. The specific days for which these models are best applicable are listed in Table S1.*

Table S1: *Table indicating the days correlating the specific equivalent circuit models that were used to model the evolution of the EIS response for AA 7075 substrates coated with the three thickness variants of 3 wt.% UFG/PEI over 100 days of immersion in a 3.5 wt.% aqueous solution of NaCl at ambient conditions as shown in Figure S4.*

coating identification	equivalent circuit model	days applicable
**4—7 µm UFG/PEI**	4c	0
	4d	1
	4e	2
	4h	40—100
	4i	7—30
**11—16 µm UFG/PEI**	4d	7
	4e	14, 30, 75—100
	4f	0, 21
	4g	1—2, 40
	4i	50
**20—30 µm UFG/PEI**	4a	0, 2—100
	4b	1

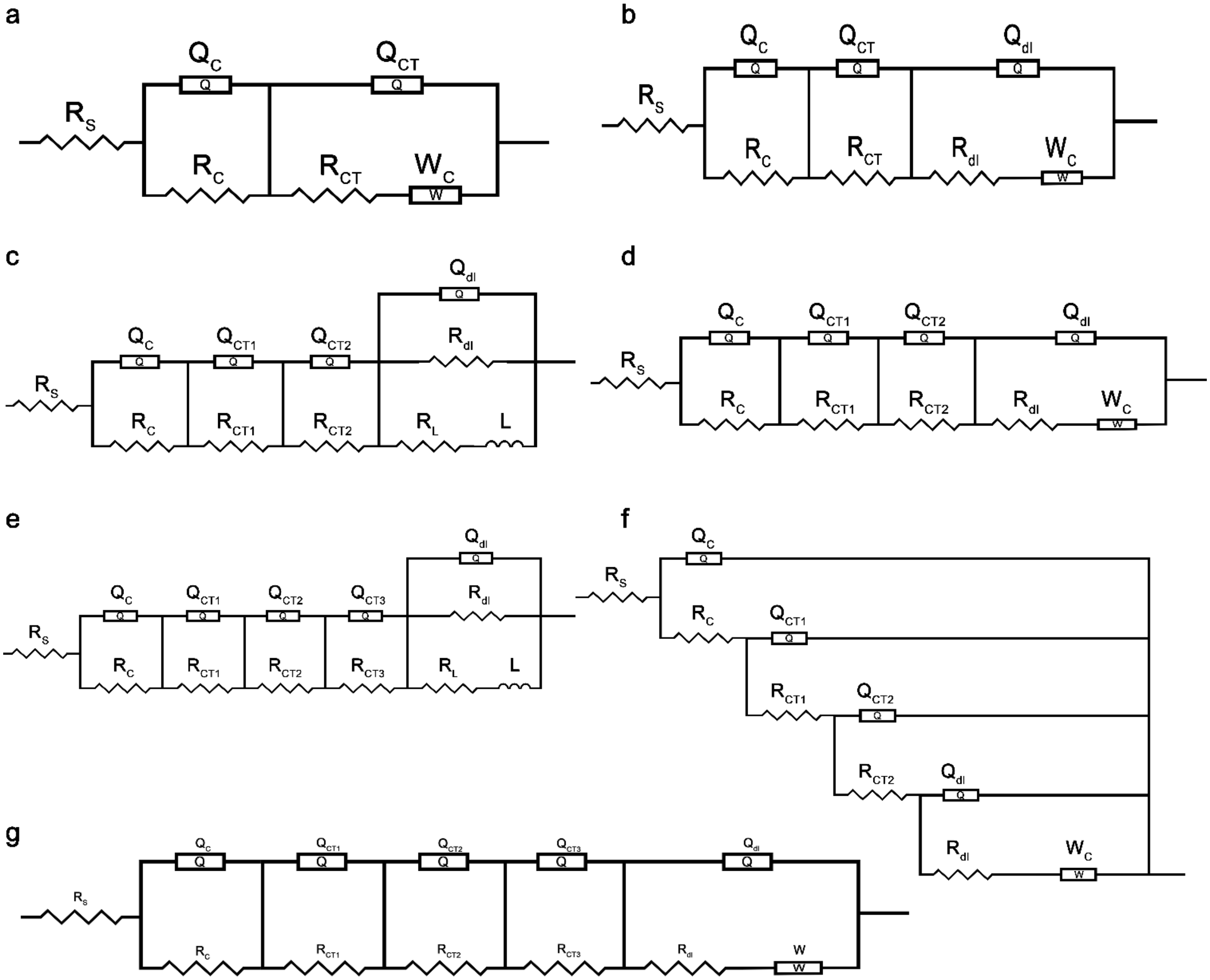

Figure S5: Equivalent circuits modeling the evolution of the impedance response of AA 7075 substrates coated with 4—7 µm Si-PEI, 11—16 µm Si-PEI, and 20—30 µm Si-PEI then immersed in a 3.5 wt.% aqueous solution of NaCl for 100 days. *a) represents two Randles cells in series with a Warburg diffusion element; b) depicts three Randles cells in series with a Warburg diffusion element; c) portrays three Randles cells in series with an inductor; d) illustrates four Randles cells in series with a Warburg diffusion element; e) shows four Randles cells in series with an inductor; f) displays four Randles cells in parallel with a Warburg diffusion element; and g) demonstrates five Randles cells in series with a Warburg diffusion element. The specific days for which these models are best applicable are listed in Table S2.*

Table S2: *Table indicating the days correlating the specific equivalent circuit models that were used to model the evolution of the EIS response for AA 7075 substrates coated with the three thickness variants of Si-PEI over 100 days of immersion in a 3.5 wt.% aqueous solution of NaCl as shown in Figure S5.*

coating identification	equivalent circuit model	days applicable
**4—7 µm Si-PEI**	5c	2
	5d	14
	5e	40,75
	5f	7
	5g	0—1, 21—30, 50, 100
**11—16 µm Si-PEI**	5b	0, 40
	5d	1—7, 50
	5g	14—30, 75—100
**20—30 µm Si-PEI**	5a	0—100

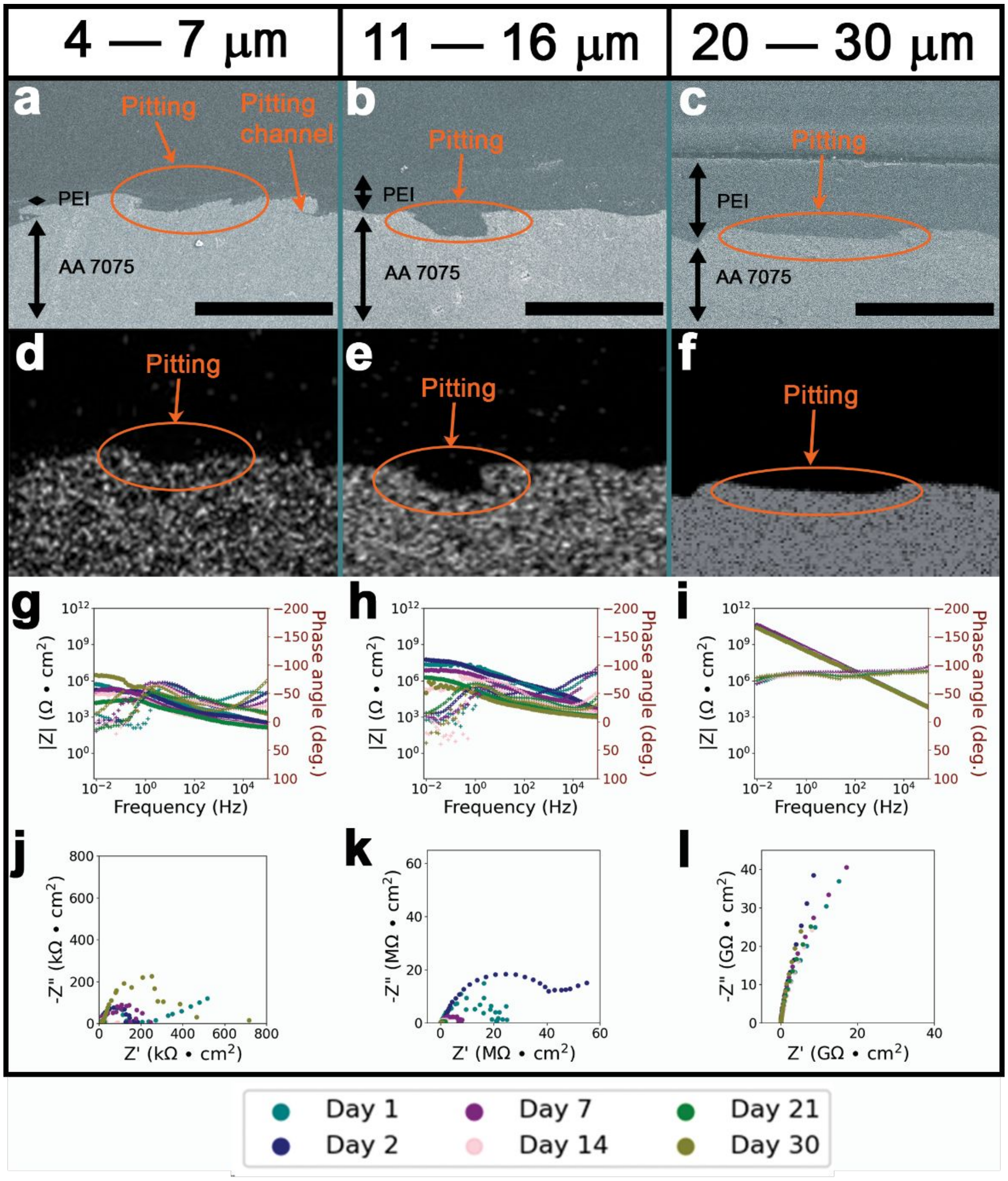

Figure S6: Corrosion performance of varying thicknesses of PEI coatings after 30 days of immersion in 3.5 wt.% aqueous solutions of NaCl at an elevated temperature of 70 °C. *Post-exposure SEM cross-sectional view of a) 4—7 μm PEI; b) 11—16 μm PEI; and c) 20—30 μm PEI on AA 7075 substrates after 30 days of immersion in 3.5 wt.% aqueous solutions of NaCl at an elevated temperature of 70 °C. The scale-bars correspond to 50 μm. Aluminum EDS maps of AA 7075 substrates coated with d) 4—7 μm PEI; e) 11—16 μm PEI; and f) 20—30 μm PEI. Bode plots corresponding to AA 7075 substrates coated with g) 4—7 μm PEI; h) 11—16 μm PEI; and i) 20—30 μm PEI. Nyquist plots for j) 4—7 μm PEI; k) 11—16 μm PEI; and l) 20—30 μm PEI monitored across 30 days of thermal immersion exposure in 3.5 wt.% aqueous solutions of NaCl at 70 °C.*

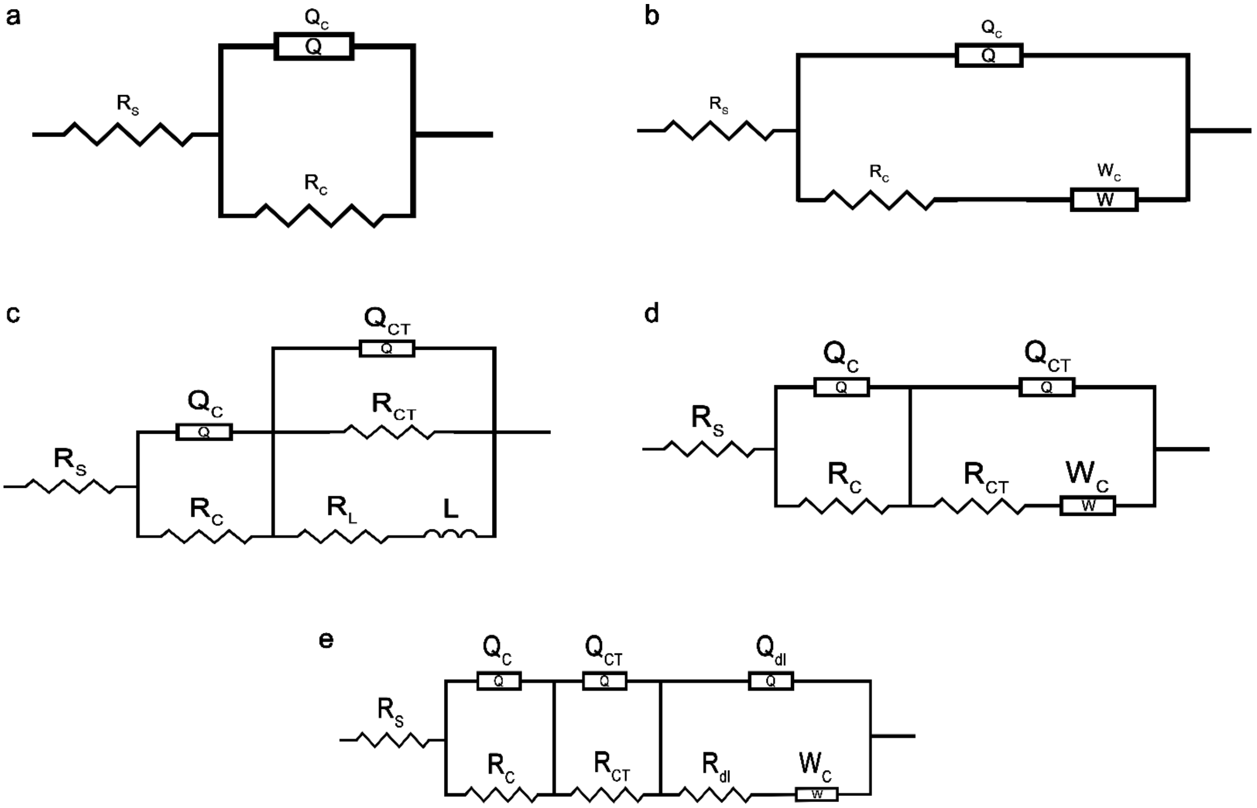

Figure S7: Equivalent circuits modeling the evolution of the impedance response of AA 7075 substrates with varying thicknesses of UFG/PEI coatings on AA 7075. *Equivalent circuits modeling the evolution of the impedance response of AA 7075 substrates coated with 4—7 µm 3 wt.% UFG/PEI, 11—16 µm 3 wt.% UFG/PEI, and 20—30 µm 3 wt.% UFG/PEI then immersed in a 3.5 wt.% aqueous solution of NaCl for 30 days at 70 °C; a) represents a single Randles cell; b) depicts a single Randles cell with a Warburg diffusion element; c) portrays one Randles cell in series with an inductor; d) illustrates two Randles cells in series with a Warburg diffusion element; and e) displays three Randles cells in series with a Warburg diffusion element. The specific days for which these models are best applicable are listed in Table S3.*

Table S3: *Days correlating the specific equivalent circuit models that were used to model the evolution of the EIS response for AA 7075 substrates coated with the three thickness variants of 3 wt.% UFG/PEI over 30 days of immersion in a 3.5 wt.% aqueous solution of NaCl at 70 °C as shown in Figure S7.*

coating identification	equivalent circuit model	days applicable
**4—7 µm UFG/PEI**	7c	0
	7d	1—30
**11—16 µm UFG/PEI**	7b	1, 7—14
	7d	2, 21—30
	7e	0
**20—30 µm UFG/PEI**	7a	0—30

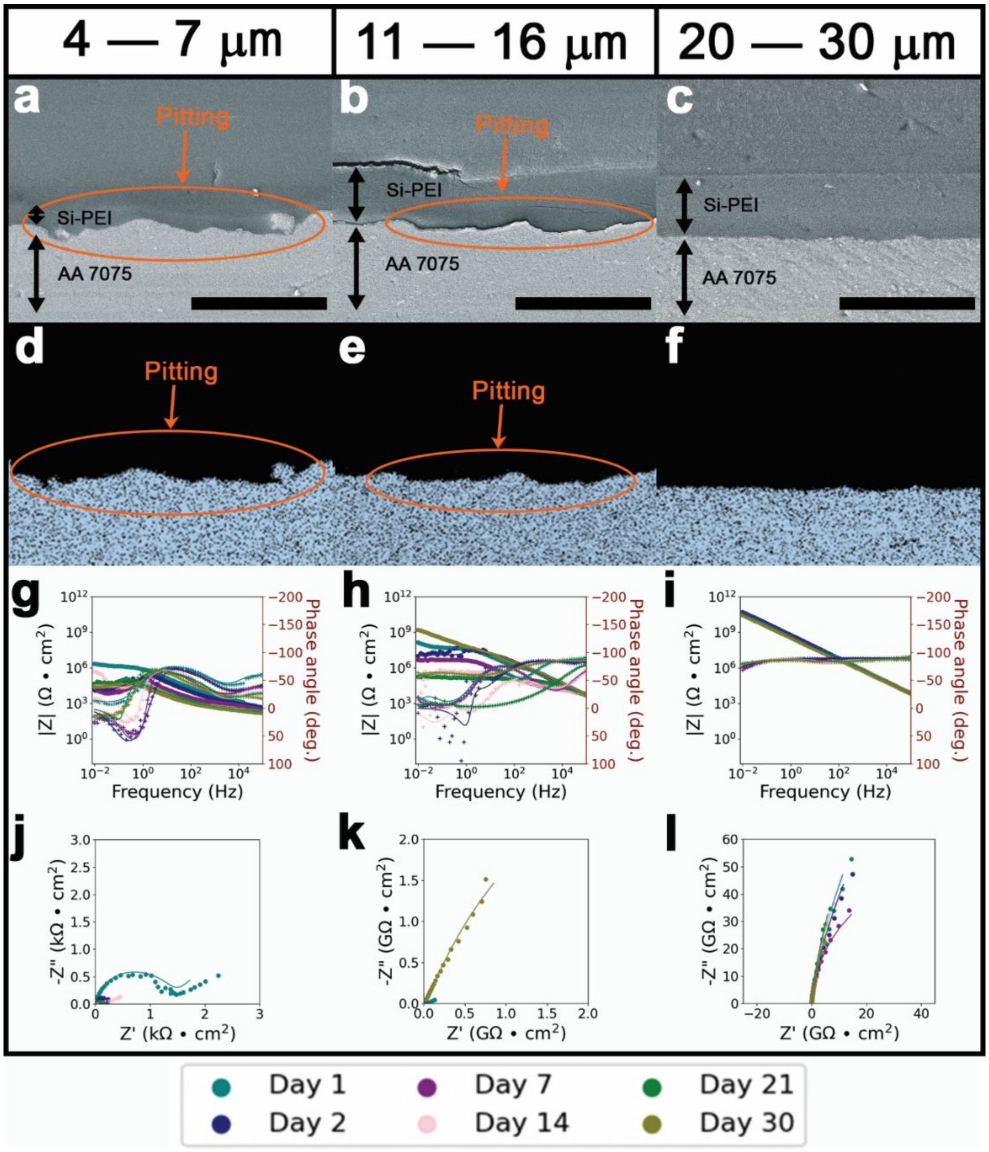

Figure S8: Corrosion performance of varying thicknesses of Si-PEI coatings after 30 days of immersion in 3.5 wt.% aqueous solutions of NaCl at an elevated temperature of 70 °C. *Post-exposure SEM cross-sectional view of a) 4—7 µm Si-PEI; b) 11—16 µm Si-PEI; and c) 20—30 µm Si-PEI on AA 7075 substrates after 30 days of thermal exposure to 3.5 wt.% aqueous solutions of NaCl at 70 °C. The scale-bars correspond to 50 µm. Aluminum EDS maps of AA 7075 substrates coated with d) 4—7 µm Si-PEI; e) 11—16 µm Si-PEI; and f) 20—30 µm Si-PEI. Bode plots corresponding to AA 7075 substrates coated with g) 4—7 µm Si-PEI; h) 11—16 µm Si-PEI; and i) 20—30 µm Si-PEI. Nyquist plots for j) 4—7 µm Si-PEI; k) 11—16 µm Si-PEI; and l) 20—30 µm Si-PEI monitored across 30 days of thermal exposure to 3.5 wt.% aqueous solutions of NaCl at 70 °C. After 30 days of immersion in 3.5 wt.% brine at elevated temperatures, the 11—16 µm Si-PEI coating underwent significant swelling (Fig. S8b) that was uniformly distributed, and adhesion improved (Table 2). The Bode plot of the 11—16 Si-PEI coated aluminum substrate exposed to thermal conditions (Fig. S8h) demonstrated instability over the 30 day immersion study resulting in an overall increase in impedance by an order of magnitude. The variable electrochemical performance is particularly apparent in the corresponding Nyquist plots (Figure 4k and Figure S8k), which illuminate stark differences in the radii of relative capacitive loops (see also Fig. S5, Fig. S10, and Table S2).*

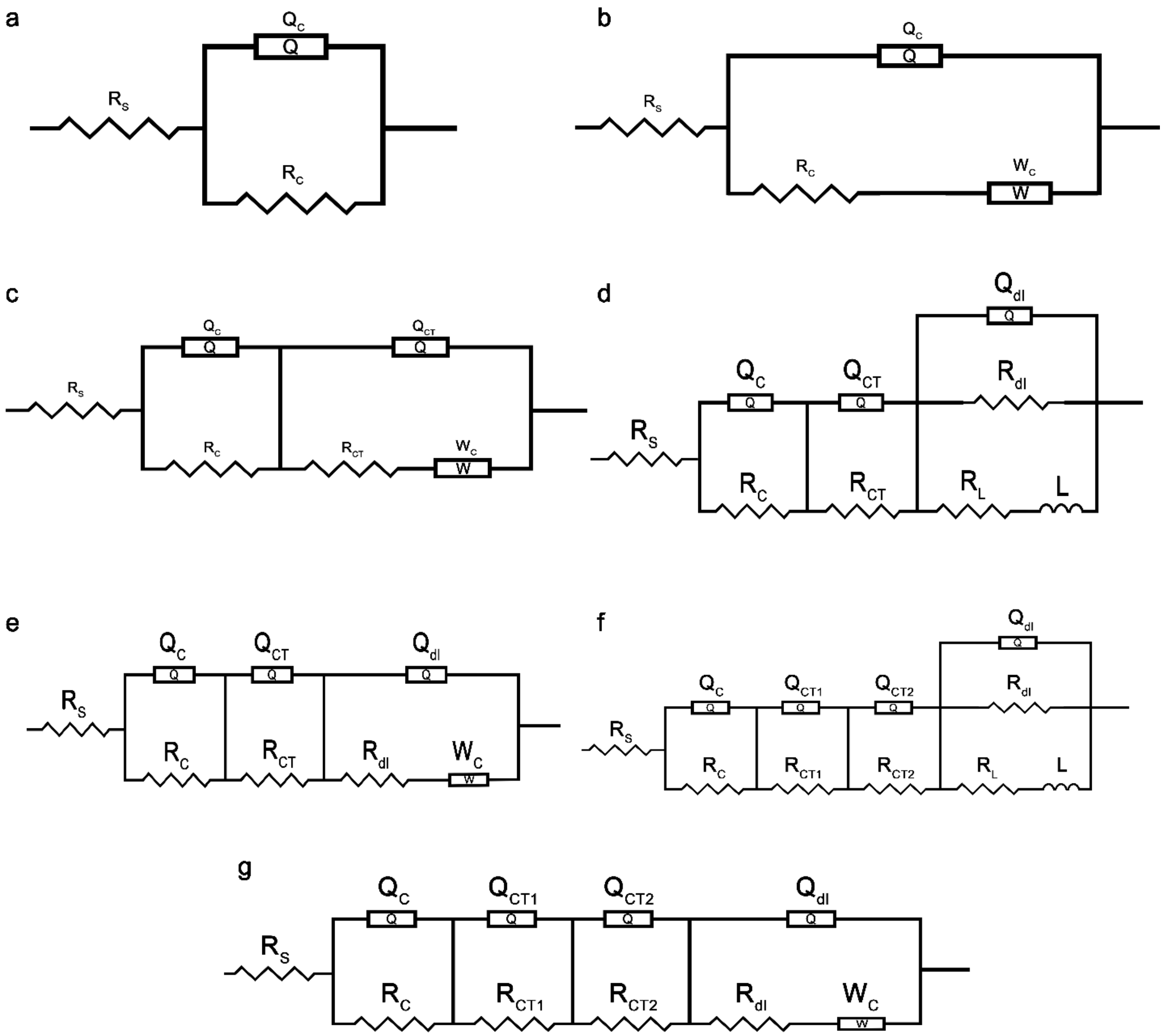

Figure S9: Equivalent circuits modeling the evolution of the impedance response of AA 7075 substrates coated with 4—7 µm Si-PEI, 11—16 µm Si-PEI, and 20—30 µm Si-PEI then immersed in a 3.5 wt.% aqueous solution of NaCl for 30 days at 70 °C. *a) represents a single Randles cell; b) depicts a single Randles cell with a Warburg diffusion element; c) portrays two Randles cells in series with a Warburg diffusion element; d) illustrates two Randles cells in series with an inductor; e) shows three Randles cells in series with a Warburg diffusion element; f) displays three Randles cells in series with an inductor; and g) demonstrates four Randles cells in series with a Warburg diffusion element. The specific days for which these models are best applicable are listed in Table S4.*

Table S4: *Table indicating the days correlating the specific equivalent circuit models that were used to model the evolution of the EIS response for AA 7075 substrates coated with the three thickness variants of Si-PEI over 30 days of immersion in a 3.5 wt.% aqueous solution of NaCl at 70 °C as shown in Figure S9.*

coating identification	equivalent circuit model	days applicable
**4—7 µm Si-PEI**	9d	14
	9e	0
	9f	2—7, 21—30
	9g	1
**11—16 µm Si-PEI**	9a	0
	9d	14
	9f	2—7, 21
	9g	1, 30
**20—30 µm Si-PEI**	9b	0—2
	9c	7—14
	9e	21—30

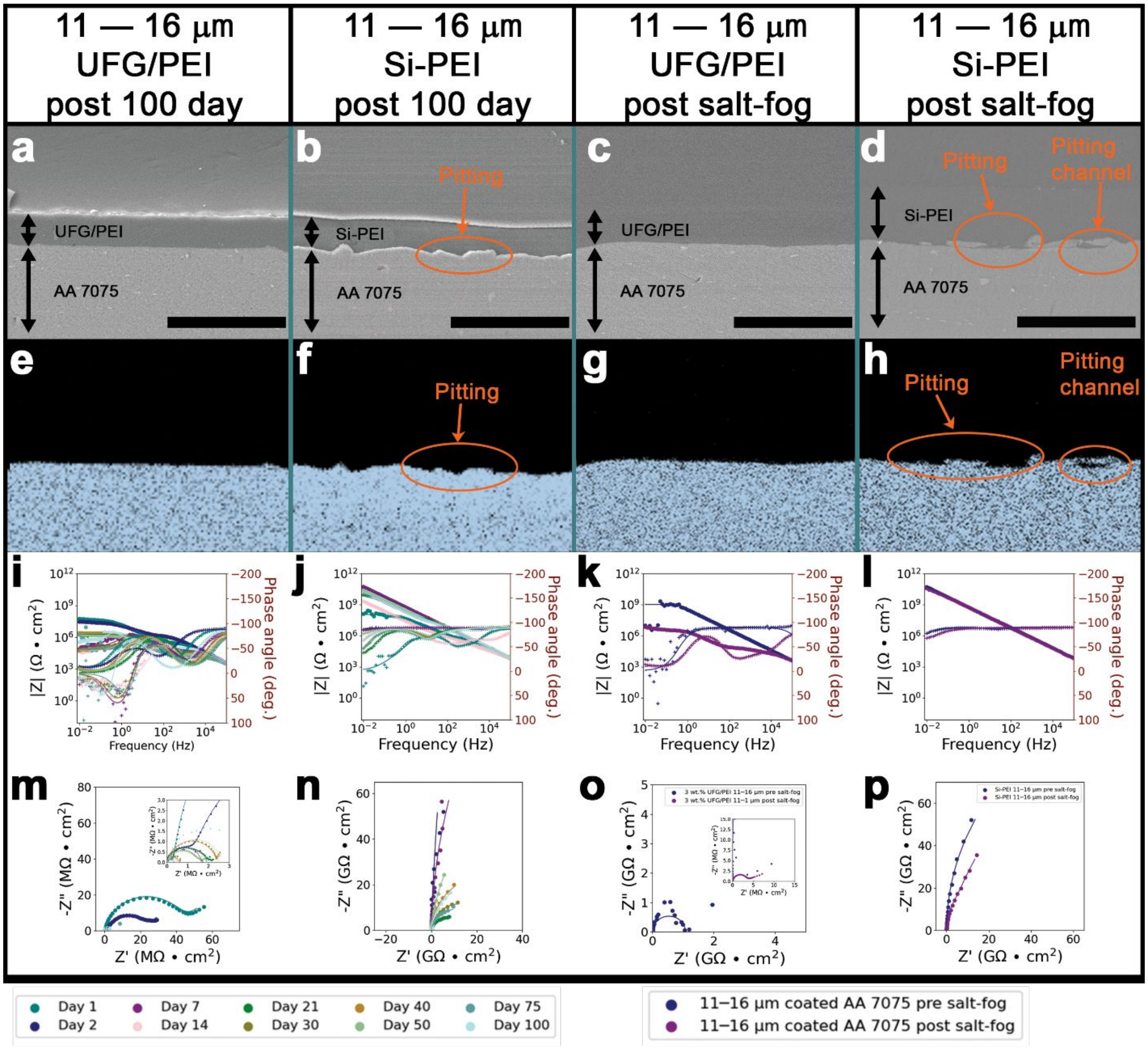

Figure S10: Corrosion performance of intermediate 11—16 µm thicknesses of UFG/PEI and Si-PEI coatings on AA 7075 after 30 days of immersion in 3.5 wt.% aqueous solutions of NaCl at an elevated temperature of 70 °C. *Post-exposure SEM cross-sectional view of a) 11—16 µm 3 wt.% UFG/PEI; and b) 11—16 µm SI-PEI on AA 7075 substrates after 100 days of exposure to 3.5 wt.% aqueous solutions of NaCl. Post-exposure SEM cross-sectional view of c) 11—16 µm 3 wt.% UFG/PEI; and d) 11—16 µm Si-PEI on AA 7075 substrates after 30 days of ASTM B-117 salt-fog exposure to 5 wt.% aqueous solutions of NaCl. The scale-bars correspond to 50 µm. Aluminum EDS maps of AA 7075 substrates coated with e) 11—16 µm 3 wt.% UFG/PEI; and f) 11—16 µm Si-PEI after 100 days of exposure to 3.5 wt.% aqueous solutions of NaCl. Aluminum EDS maps of AA 7075 substrates coated with g) 11—16 µm 3 wt.% UFG/PEI; and h) 11—16 µm SI-PEI after 30 days of ASTM B-117 salt-fog exposure to 5 wt.% aqueous solutions of NaCl. Bode plots corresponding to AA 7075 substrates coated with i) 11—16 µm 3 wt.% UFG/PEI; and j) 11—16 µm Si-PEI over 100 days of immersion in 3.5 wt.% aqueous NaCl solutions at ambient conditions. Bode plots corresponding to AA 7075 substrates coated with k) 11—16 µm 3 wt.% UFG/PEI; and l) 11—16 µm Si-PEI after 30 days of ASTM B-117 salt-fog exposure in 5 wt.% aqueous NaCl. Nyquist plots for m) 11—16 µm 3 wt.% UFG/PEI; and n) 11—16 µm Si-PEI coated AA 7075 substrates monitored across 100 days of exposure to 3.5 wt.% aqueous solutions of NaCl. Nyquist plots for o) 11—16 µm 3 wt.% UFG/PEI; and p) 11—16 µm Si-PEI coated AA 7075 substrates monitored across 30 days of ASTM B-117 salt-fog exposure to 5 wt.% aqueous solutions of NaCl.*

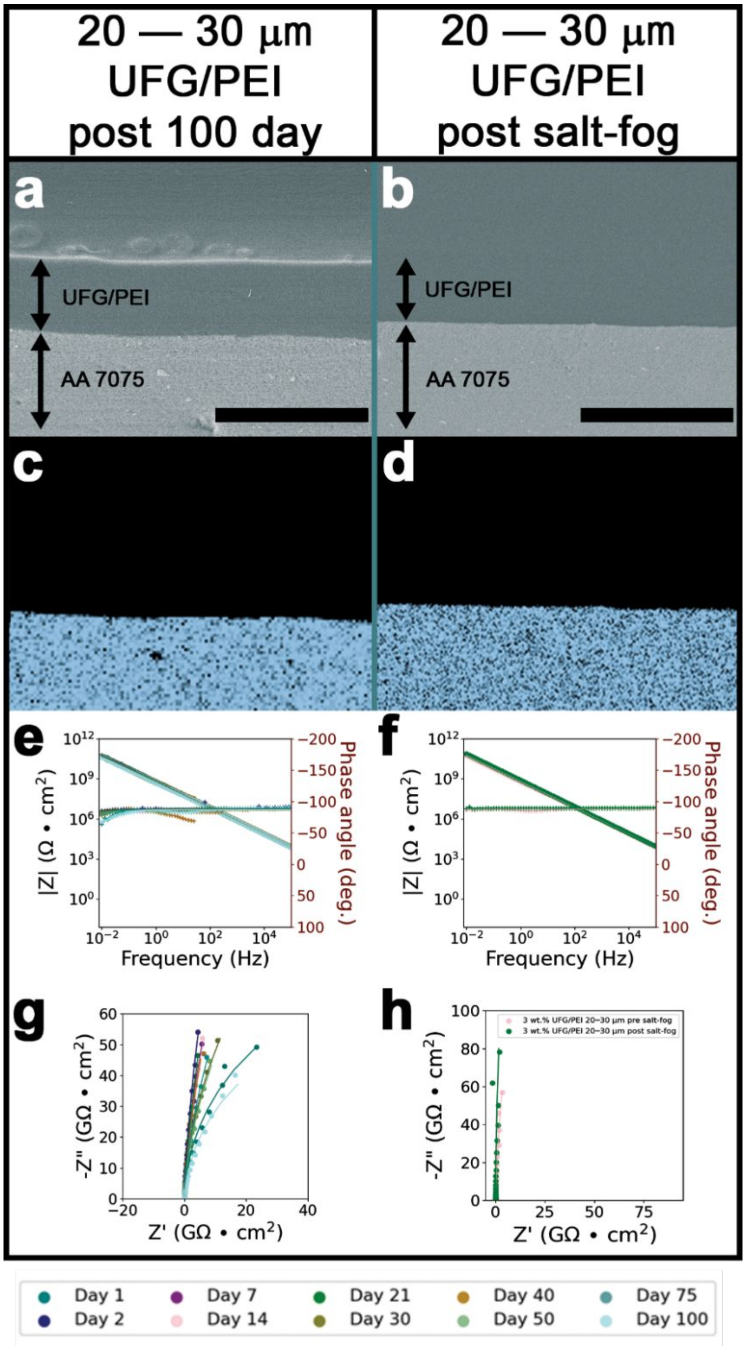

Figure S11: Corrosion performance of 20—30 μm thicknesses of UFG/PEI-coated AA 7075 after 100 days of immersion in 3.5 wt.% aqueous solutions of NaCl and ASTM B-117 salt-fog exposure for 30 days. *Post-exposure SEM cross-sectional view of a) 20—30 μm 3 wt.% UFG/PEI on AA 7075 substrate after 100 days of exposure to 3.5 wt.% aqueous NaCl solution. Post-exposure SEM cross-sectional view of b) 20—30 μm 3 wt.% UFG/PEI on AA 7075 substrate after 30 days of ASTM B-117 salt-fog exposure to a 5 wt.% aqueous solution of NaCl. The scale-bars correspond to 50 μm. Aluminum EDS map of AA 7075 substrates coated with c) 20—30 μm 3 wt.% UFG/PEI after 100 days of exposure to 3.5 wt.% aqueous brine solution. Aluminum EDS map of AA 7075 coated with d) 20—30 μm 3 wt.% UFG/PEI after 30 days of ASTM B-117 salt-fog exposure to a 5 wt.% aqueous solution of NaCl. Bode plot corresponding to AA 7075 coated with e) 20—30 μm 3 wt.% UFG/PEI over 100 days of immersion in 3.5 wt.% aqueous NaCl solution at ambient conditions. Bode plot corresponding to an AA 7075 substrate coated with f) 20—30 μm 3 wt.% UFG/PEI after 30 days of ASTM B-117 salt-fog exposure in 5 wt.% aqueous NaCl. Nyquist plot for g) 20—30 μm 3 wt.% UFG/PEI coated AA 7075 monitored across 100 days of exposure to 3.5 wt.% aqueous NaCl. Nyquist plot for h) 20—30 μm 3 wt.% UFG/PEI coated AA 7075 monitored across 30 days of ASTM B-117 salt-fog exposure to a 5 wt.% aqueous solution of NaCl.*

References

1. Davidson, R. D. *et al.* Tortuosity but Not Percolation: Design of Exfoliated Graphite Nanocomposite Coatings for Extended Corrosion Protection of Aluminum Alloys. *ACS Appl Nano Mater* 2, 3100–3116 (2019).
